# Diversity–Elevation Relationships of Vascular Plants in Austral Temperate Ecosystems Are Strata Dependent

**DOI:** 10.1002/ece3.72515

**Published:** 2025-12-19

**Authors:** T. Michelt, D. Craven, V. Peréz‐Tello, S. D. H. Irl

**Affiliations:** ^1^ Biogeography and Biodiversity Lab, Institute of Physical Geography Goethe‐University Frankfurt Frankfurt am Main Germany; ^2^ GEMA Center for Genomics, Ecology & Environment Universidad Mayor Santiago Chile; ^3^ Data Observatory Foundation ANID Technology Center Santiago Chile

**Keywords:** diversity indices, elevation gradient, environmental filtering, forest strata, land‐use effects, mountain ecosystems, southern South America, temperate rainforest

## Abstract

Using elevational gradients as natural laboratories to examine patterns and drivers of biodiversity has a long history in biogeography and ecology. Across mountain ecosystems globally, diversity commonly decreases with increasing elevation. However, anthropogenic disturbances—particularly those at lower elevations—may alter the diversity–elevation relationship. Here, we examined elevational diversity patterns, community composition, and the effect of disturbances on the diversity–elevation relationship of vascular plants in Hornopirén (Chile). Using a total of 44 plots along two transects, we assessed species diversity in undisturbed habitats (800–1500 m) and in undisturbed and disturbed habitats (300–1500 m) for overstorey and understorey vegetation. We found that species richness of overstorey and understorey vegetation decreased monotonically with elevation, but that of understorey vegetation responded less markedly to elevation. While the Simpson diversity‐elevation relationship for understorey vegetation showed a hump‐shaped relationship, that of overstorey vegetation decreased monotonically with elevation. Our results are consistent with the idea that decreasing temperature along the elevation gradient likely operated as an environmental filter on overstorey diversity, whereas understorey vegetation was more decoupled from temperature in forested habitats. In the alpine scrub (> 1300 m), variation in Simpson diversity, species evenness, and species composition suggests that biotic interactions potentially shape plant community structure to a greater extent than abiotic conditions. Our results also indicate that the shifted shape of the diversity–elevation relationships and altered species composition when including both undisturbed and disturbed habitats might be associated with anthropogenic disturbances. This suggests that human disturbances fundamentally alter natural processes generating diversity–elevation relationships. We emphasize the importance of assessing biodiversity inside and outside protected areas to detect potential shifts in local biodiversity patterns associated with anthropogenic land use and argue that sustainable management and conservation strategies are essential for mitigating further human impacts on these unique ecosystems.

## Graphical abstract

We investigated diversity patterns of vascular plants of overstorey and understorey vegetation separately per stratum and the influence of anthropogenic disturbances along two elevational transects with contrasting slope aspects in a temperate rainforest and alpine scrub in southern Chile. We found that overstorey diversity decreased monotonically along elevation due to environmental filtering, whereas understorey diversity was more decoupled from elevation and responded less markedly, most likely due to the climatic buffering of the tree and shrub layers. Above the treeline, diversity patterns of alpine vegetation suggested that biotic interactions and local factors like slope aspect and topography may be increasingly important in shaping abundance distributions and species composition of communities. Our results also suggest that land use alters the shape of diversity–elevation relationships.

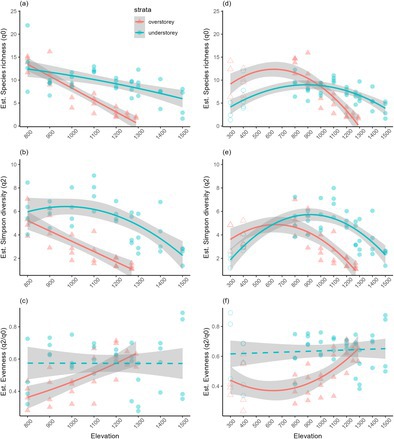

## 1 Introduction

Deciphering patterns and identifying drivers of the heterogeneous distribution of life on Earth are fundamental questions of biogeography and ecology (Bonpland and von Humboldt 1807; Rosenzweig 1995; Gaston 2000). Since the 19th century, geologists and geographers such as Charles Darwin, Alexander von Humboldt, and Alfred R. Wallace observed changes in species composition and decreases in species richness with increasing elevation or latitude (Wallace 1878; Lomolino 2001; McCain and Grytnes 2010). While only 10% of the Earth's surface is covered by mountains, they host approximately 25% of all terrestrial species, a third of all terrestrial plant species and a half of global biodiversity hotspots (Körner 2004; Chape 2008; Körner et al. 2017; Antonelli et al. 2018; Körner and Spehn 2019). Thus, mountain ecosystems provide a compelling template for examining how biodiversity responds to elevation, which is used as a proxy for climatic conditions (McCain and Grytnes 2010; Lomolino 2001; Rahbek et al. 2019; Bhatta et al. 2021; Vetaas et al. 2025). Over relatively short distances, shifts in diversity along elevation gradients frequently parallel those found across broad‐scale gradients (Körner 2000; Vetaas 2021). Along elevational gradients, biodiversity often declines because the distributions of plants are constrained principally by water availability or temperature (E. O'Brien 1998; Hawkins et al. 2003; Currie et al. 2004; Sommer et al. 2010; Vetaas et al. 2025). Thus, warm and wet conditions favor greater biodiversity by facilitating diversification and providing more niche space (McCain 2009; McCain and Grytnes 2010; Antonelli et al. 2018; Allen et al. 2002). Local climate also can be modulated by a mountain's topographical features, such as slope aspect, which may increase (or decrease) insolation, precipitation, or wind, thereby influencing species richness and composition (Ferreyra et al. 1998; Grytnes and McCain 2013; Irl et al. 2015; Ezcurra and Gavini 2020). Besides abiotic conditions, biotic interactions are also thought to influence species richness (McCain and Grytnes 2010); competition is expected to decrease species richness in productive environments through competitive exclusion, whereas facilitative interactions are expected to enhance richness in stressful environments, as plants potentially create microhabitat conditions that permit other species to persist in otherwise harsh environmental conditions (Terborgh and Weske 1975; Bertness and Callaway 1994; van der Heijden et al. 1998; Körner 2004; Badano and Cavieres 2006; Cavieres et al. 2016). Shifts in environmental conditions, topographic features, and biotic interactions therefore jointly shape diversity–elevation relationships, which can vary from monotonic decreasing (i.e., diversity highest at lower elevations) to hump‐shaped (i.e., diversity highest at midelevations), low plateau (i.e., highest diversity at lower and midelevations, and decreasing diversity towards upper elevations), or multimodal (i.e., idiosyncratic peaks of diversity) (McCain and Grytnes 2010).

**FIGURE 1:**
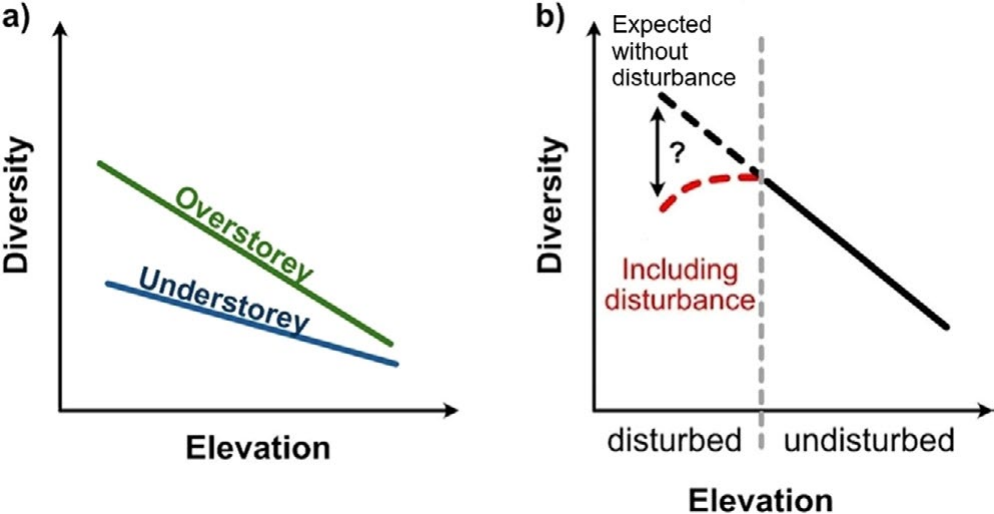
Conceptual figure detailing expected diversity–elevation relationships for different forest strata (a) and for elevation gradients with disturbed habitats at lower elevations (b). Because overstorey diversity is more directly coupled with climate, it will respond more strongly to elevation, while understorey diversity is only weakly linked with climate, leading to a less pronounced or ambiguous reaction to elevation. Including disturbed habitats at lower elevations will alter the shape of diversity–elevation relationships from monotonically decreasing to a hump‐shaped relationship, as human activities will lower diversity at lower elevations. We expect these patterns to be similar across Hill numbers 0 and 2.

Across most biomes, mountain ecosystems are dominated by forests across large parts of the elevational gradient (Körner 2004). The structural heterogeneity of forest canopies often creates substantially different microclimates for understorey species, buffering the impacts of climate compared to overstorey conditions (Körner 2007; Zellweger et al. 2020). Consequently, the association of plants with a certain forest stratum might influence responses to local factors such as thermal conditions, light availability, aspect and topography, community interactions, or disturbances (Ohlemüller and Wilson 2000; Pausas and Austin 2001; Grytnes et al. 2006; Wang et al. 2009). Therefore, increasing climatic harshness with elevation (i.e., lower temperatures, stronger winds and greater exposure to rain) may affect overstorey vegetation more strongly than understorey vegetation. Evaluating forest strata separately can reveal the contrasting patterns and drivers of plant diversity for each stratum (Figure 1a).

Species richness is the most commonly used measure of diversity (Whittaker 1972; Magurran 2004; Gotelli and Colwell 2011; Morris et al. 2014; Chao and Jost 2012; Chao et al. 2014, Roswell et al. 2021). However, it is also well recognized that species richness is a statistically problematic index and provides limited insights to community structure. First, the observed number of species depends strongly on sample effort (i.e., sample size, individuals, and sample area). It usually underestimates the true species richness of a community and as samples are finite, it is crucial to develop and use methods that estimate richness more accurately (so‐called *sampling problem*, Chao et al. 2014). Without standardizing sampled species richness, diversity patterns can be significantly biased (Rahbek 1995; McCain and Grytnes 2010; Gotelli and Colwell 2011; Maurer and McGill 2011; Chao et al. 2014; Roswell et al. 2021). Second, species richness does not account for the relative abundances of the species in a community and is as sensitive to rare species as it is to common ones (so‐called *abundance problem*, Chao et al. 2014). In the case of communities with the same numbers of species, diversity should be higher in the assemblage with a more equal relative abundance distribution. Hence, the sample dominated by one or a few species is less diverse than species richness alone would indicate (Pielou 1975; Maurer and McGill 2011; Chao et al. 2014; Roswell et al. 2021). In order to account for these issues, an estimation of Hill numbers (*q* = 0 for species richness, *q* = 1 for Shannon diversity, *q* = 2 for Simpson diversity, Hill 1973) through the integration of sample size‐based and sample coverage‐based rarefaction and extrapolation facilitates fair comparisons of incomplete samples and incorporates species relative abundances (Chao and Jost 2012; Chao et al. 2014; Roswell et al. 2021). Hill numbers are a mathematically unified family of diversity indices, and sensitivity to relatively rare species decreases with an increasing order *q*, as *q* = 1 and *q* = 2 emphasize common and dominant species, respectively. Furthermore, the relation between species richness and Simpson diversity enables an assessment of evenness with the q2/q0 ratio, or comparing slopes of the modeled q0‐elevation and q2‐elevation relationships, to gain a deeper insight into community structure (Hill 1973; Chao et al. 2014; Morris et al. 2014). Applying estimated Hill numbers to elevational gradients might shed new insights into diversity–elevation relationships, because potential species pools per elevational band show a directional decrease towards higher elevations as a result of the smaller area of elevational bands at higher elevations compared to low elevations (Rosenzweig 1995; Körner 2000; Romdal and Grytnes 2007; McCain and Grytnes 2010).

Yet, mountain biodiversity is highly endangered worldwide (Körner 2004) largely due to growing human pressures (Elsen et al. 2020). While describing biodiversity patterns of mountain ecosystems and identifying their underlying drivers is of fundamental importance (McCain and Grytnes 2010), it is also crucial for effective conservation strategies to assess how human activities have impacted biodiversity in mountain ecosystems (Peters et al. 2019; Joelson et al. 2025). Anthropogenic impacts in mountain ecosystems are a critical factor for diversity–elevation relationships (Costa et al. 2023) as they are typically stronger towards lower elevations (Nogués‐Bravo et al. 2008; Elsen et al. 2020). Their concentration at lower elevations may reflect differences in land‐use history or disturbance type, as deforestation, habitat fragmentation, and land‐use change are more frequent at lower elevations, while cattle grazing or anthropogenic fire are more common at higher elevations (Körner 2004; Nogués‐Bravo et al. 2008; Newbold et al. 2015; Elsen et al. 2018, 2020). Disturbances by free‐ranging livestock (Hobbs 2001; Floyd et al. 2003; Wassie et al. 2009) and selective logging (Rüger et al. 2007; Farwig et al. 2008) may affect species diversity and forest dynamics in mountain ecosystems. The impacts of such disturbances, particularly on forest structure and regeneration, are amplified if they overlap in space and occur continuously (Ramírez‐Marcial 2003). Yet, the impacts of anthropogenic disturbances on the shape of diversity‐elevation relationships for plants remain largely uncertain (Nogués‐Bravo et al. 2008, but see Peters et al. 2019; Monge‐González et al. 2020; Costa et al. 2023).

Research on diversity–environment relationships and responses to human activities is geographically biased, and is largely concentrated in the Northern Hemisphere within the tropical, northern temperate forest and boreal ecoregions (Guo et al. 2013; Murphy and Romanuk 2014; Grantham et al. 2020; Costa et al. 2023). There is a limited number of studies on local elevational patterns of plant species diversity in southern South America (e.g., Becerra 2016; López‐Angulo et al. 2018; or Arroyo et al. 2024 for the Chilean Andes), as well as studies on disturbance‐mediated biodiversity loss in austral temperate rainforests, where plant diversity is principally threatened by land use and fragmentation (Echeverría et al. 2007; Guo et al. 2013; Murphy and Romanuk 2014; Newbold et al. 2016; Arroyo 2023). Due to its geographic and climatic isolation, the Valdivian temperate rainforests are considered a biogeographic island (Armesto et al. 1997). Thus, it is essential to document and disentangle diversity patterns of this unique and threatened ecosystem and to examine its sensitivity to anthropogenic pressures within and outside of protected areas, as a baseline for effective conservation policies (Elsen et al. 2018; Joelson et al. 2025).

In the present study, we investigated diversity patterns of vascular plants and the influence of anthropogenic disturbances along two elevational transects with contrasting slope aspects in a temperate rainforest and alpine scrub in southern Chile. First, we assessed plant species diversity and community composition of each stratum (overstorey and understorey) separately along the undisturbed elevational gradient. We hypothesized (i) that plant species diversity of the overstorey stratum would decrease monotonically with increasing elevation principally due to the decline in temperature with elevation but similar levels of water availability along the elevational gradient (Hawkins et al. 2003; Evans et al. 2005; McCain 2009; McCain and Grytnes 2010; Figure 1a), while (ii) species diversity of the understorey stratum would respond less markedly to elevation‐driven changes in abiotic conditions, because overstorey vegetation might buffer understorey vegetation from decreasing temperature with elevation (Körner 2007; Wang et al. 2009; Figure 1a). Second, when including disturbed forests at lower elevations, we expected that the shape of the diversity–elevation relationship would shift from monotonically decreasing to hump‐shaped, as land uses such as timber and fuelwood harvesting and cattle grazing likely lower species diversity at lower elevations (Newbold et al. 2015; Peters et al. 2019; Figure 1b).

Lastly, we expected that plant species composition would shift with elevation and would differ between undisturbed and disturbed forests (Peters et al. 2019; Monge‐González et al. 2020), and between slope aspects (Ferreyra et al. 1998; Ezcurra and Gavini 2020).

## 2 Material and Methods

### 2.1 Study Area

**FIGURE 2:**
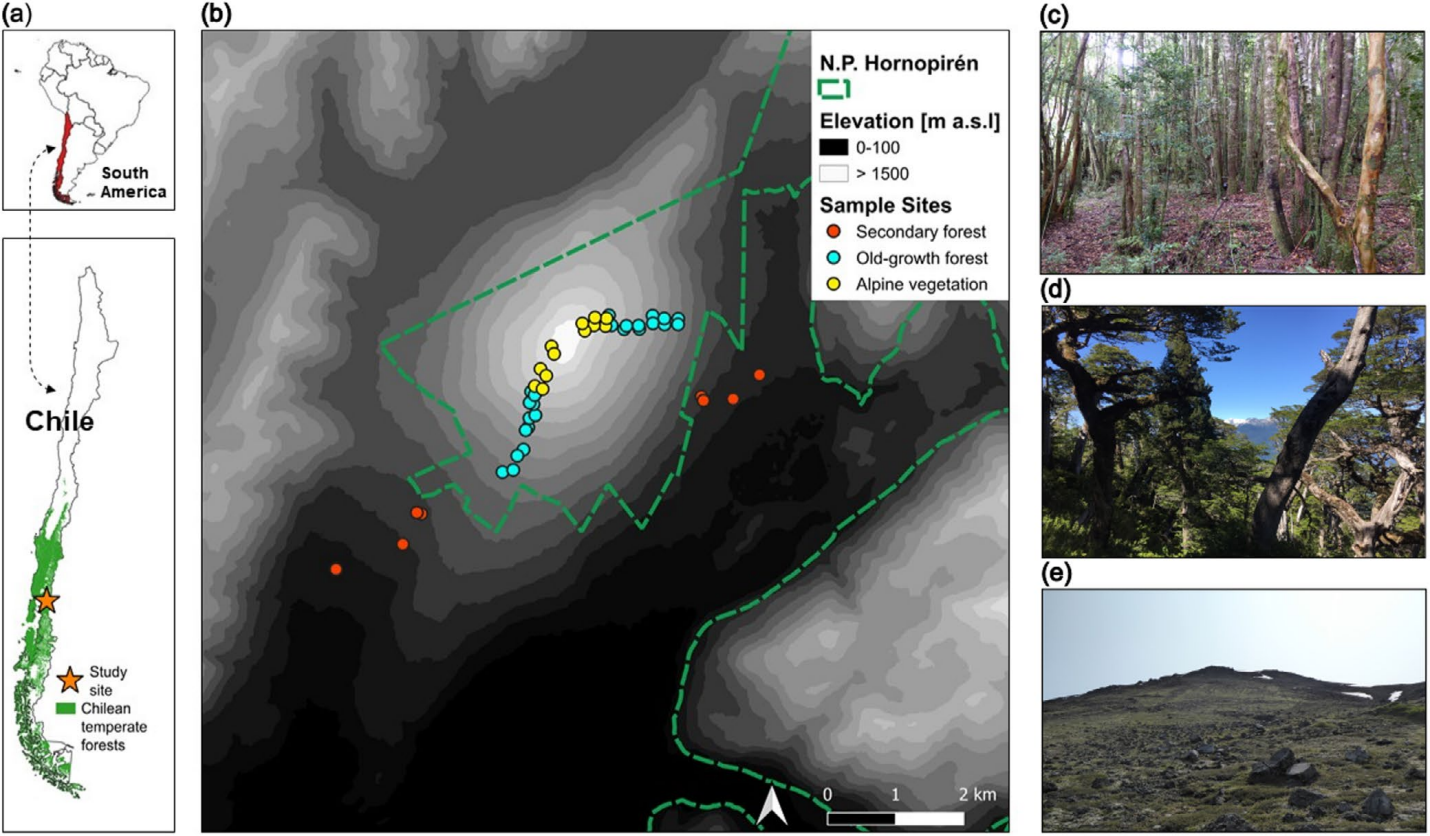
Map of study site in Hornopirén, Chile, South America (a). Green color indicates distribution of temperate forests in Chile (Luebert and Pliscoff 2006). In (b) points (red: Disturbed secondary forest, turquoise: Undisturbed old‐growth forest, yellow: Alpine vegetation) indicate sampling locations (plots of 20 m × 20 m) along the north‐eastern (Transect 1) and south‐western (Transect 2) slopes of the Hornopirén volcano (digital elevation model from MBN 2019; border of National Park Hornopirén from MMA 2025). Images in (c–e) show examples of the three vegetation types: Secondary forest (c), old‐growth forest (d) and alpine vegetation (e). (Images by T. Michelt 2020).

The study was performed along the southwestern and northeastern slopes of Hornopirén volcano (1572 m a.s.l.) in southern Chile (41°52′S, 72°26′W, Figure 2). Since 2018, the volcano has been part of Hornopirén National Park, which was established in 1988 (CONAF 1999; Ministerio de Interior y Seguridad Pública 2018).

According to the Köppen and Geiger climate classification, the climate in the region of Hornopirén is classified as Cfb (marine west coastal climate) with an average temperature throughout the year of 10°C at the coast, 9°C at the base of the mountains and 3°C–4°C at the top of Hornopirén volcano (CONAF 1999; Geiger 1961; Karger et al. 2018). The symmetrical cone shape of the andesitic basaltic stratovolcano Hornopirén indicates a geologically young age, infrequent eruptions and mainly lava production during the Holocene. The only significant explosive eruption is thought to have occurred in the mid‐Holocene (~5700 year. BP, ad 1950). Minor eruptions with lava flows occurred ~1500 years. BP (Watt et al. 2011). The most recent volcanic activity supposedly occurred in 1835, but corroborating evidence is lacking (Global Volcanism Program 2013). The average temperature of the warmest month (January) ranges from 15°C to 14°C, and 6°C to 7°C for the coldest month (July). Precipitation in Hornopirén National Park varies between 3000 mm y^−1^ near the coast and 5000 mm y^−1^ at higher elevations in the southeastern sector of the Park (CONAF 1999). Generally, there are three main forest types along the elevational gradient in the study area: broadleaved evergreen forest at lower elevations (*Nothofagus nitida*, Nothofagaceae; *Amomyrtus luma*, *Luma apiculata* , both Myrtaceae), mixed coniferous and broadleaved *Fitzroya cupressoides* (Cupressaceae) and *Nothofagus betuloides* dominated forests at mid elevations, and deciduous broadleaved *Nothofagus pumilio* (both Nothofagaceae) dominated forests at higher elevations (Donoso 1981). However, due to human activities such as timber harvest, fuel wood cutting or cattle grazing, the broadleaved evergreen forests at lower elevations are often degraded secondary forests (see following section). The treeline occurs at approximately 1300 m with *Nothofagus pumilio* and *N. antarctica* as the prevalent tree species. The alpine scrub mostly consists of herbaceous rosette plants such as *Senecio* spp. (Asteraceae) and low stature shrubby species, for example, *Tribeles australis* (Escalloniaceae) and *Empetrum rubrum* (Ericaceae).

### 2.2 Study Design and Data Sampling

We collected data along two transects at the south‐western windward and north‐eastern leeward slopes of Hornopirén volcano (Figure 2). Data sampling was conducted in the main vegetation period from January to March 2020.

The elevational gradient contains the following ecosystems: disturbed secondary forest (< 800 m, see below), undisturbed old‐growth forest (800 m–1270 m), and alpine scrub above the treeline (1300 m–1500 m). Using the following criteria, we identified old‐growth forests above 800 m: minimal or no visual evidence of human intervention, dominance of old‐growth species in the canopy, such as *Podocarpus nubigena* (Podocarpaceae) and shade‐intolerant to semi‐tolerant *Nothofagus* species (Veblen et al. 1996; Armesto et al. 2009), complex vertical structure with emergent pioneers, gap‐phase dynamics, fallen logs in various stages of decomposition, and a species composition that has not been significantly modified due to human impacts or large disturbances (Mosseler et al. 2003; Wirth et al. 2009; Armesto et al. 2009; Gutiérrez et al. 2009; Ponce et al. 2017).

As mentioned above, forests at lower elevations of the volcano (below approximately 700 m) have been disturbed or degraded, largely due to timber and firewood extraction and cattle grazing (T. Michelt, pers. obs.). These largely anthropogenic disturbances have created an inaccessible thicket dominated by bamboos (*Chusquea* spp., Poaceae), tree ferns (e.g., *Lophosoria quadripinnata*, Dicksoniaceae), shrubs (e.g., *Fuchsia magellanica*, Onagraceae; *Desfontainia fulgens*, Columelliaceae), and some remnant trees, between 500 m and 700 m that could not be sampled. Thus, secondary forests could only be sampled at 300 m and 400 m, which are outside of the borders of National Park Hornopirén (see Figure 2).

Along each transect, we established two plots of 20 m × 20 m at intervals of 100 m of elevation. We chose plot locations at random with a horizontal distance between plots of > 100 m to reduce spatial autocorrelation. In each plot, we recorded species identities and abundances for all vascular plants. To examine elevational diversity patterns separately per forest stratum (Körner 2007; Grytnes and McCain 2013; Peters et al. 2016), we used a nested plot design and sampled every individual with a diameter of > 1 cm at a height of 1.30 m (shrubs and trees; overstorey strata) in 20 m × 20 m plots. In each corner of each plot, we established a 1 m × 1 m quadrat and sampled all individuals of species less than 1.30 m high (herbs, juvenile shrubs and trees; understorey strata). We pooled understorey quadrats (4 × 1 m^2^) per overstorey plot. We counted corms of clonal species (e.g., *Chusquea* spp.) as individuals, and not their stems. We did not assess epiphytes that were growing on living trees or shrubs, but we recorded epiphytes rooted in the ground. We could not distinguish between individuals for cushion plant species above the treeline (e.g., *Tribeles australis*) or for species that grow in mats on the forest floor (e.g., *Philesia magellanica*, Philesiaceae). To assess abundance in an equivalent manner for such species, we used a frequency‐based method by dividing each quadrat into four subquadrats of 0.5 m × 0.5 m and counted species occurrence in one of the subquadrats as one individual. Thus, the maximum number of individuals for such species per 1 m × 1 m quadrat was four. For each species, we collected one sample and used it to confirm species identities at the Herbarium of Universidad de Concepción (Herbarium Code: CONC).

In total, we sampled 44 plots across both transects (22 in each), with four replicates per elevation level. Thirty‐six plots were located along the undisturbed part of the gradient between 800 m and 1500 m (20 plots in the forested part, four plots at the treeline at ~1270 m, 12 plots above the treeline) and eight plots were established in secondary forests at 300 m and 400 m (Figure 2). However, in the secondary forest of the north‐eastern transect, we could only establish one plot at 300 m due to forest clearcutting. We therefore sampled three plots at 400 m in the north‐eastern transect, to have the same number of replicates of secondary forest plots in both transects.

### 2.3 Species Diversity

We identified species in the field following Rodriguez et al. (2018), and later harmonized species names using the Taxonomic Name Resolution service with the R package *taxize*, whose taxonomic backbone is based on The Plant List version 1.1 (Boyle et al. 2013; iPlant Collaborative 2020).

To be able to analyze the elevation–diversity relationship while addressing the sample problem and the abundance problem, we followed Chao and Jost (2012) and Chao et al. (2014) and standardized samples by coverage and estimated diversity in terms of effective number of species for Hill numbers 0 and 2 (Hill 1973).

Standardizing by coverage is superior to sample size for comparing diversities between samples (Chao and Jost 2012). Traditional rarefaction or extrapolation to equal sample sizes does not necessarily enable fair comparisons between samples, because it does not account for the species abundance distribution of the assemblages as only sample effort is standardized, but not sample completeness. The chosen sample size might be sufficient to fully characterize the assemblage with lower diversity, but not necessarily for the more diverse assemblage (Roswell et al. 2021). Additionally, sample size‐based rarefaction and extrapolation can result in excluding large amounts of the sampled data. In contrast, standardizing by sample coverage enables comparison of samples of equal population fractions of communities, as samples of equal completeness or quality are assessed. Accordingly, coverage‐based standardization provides more robust comparisons among communities (Chao and Jost 2012; Chao et al. 2014).

We selected Hill numbers 0 and 2 to incorporate species abundances and facilitate a deeper insight into community structure, as species richness (*q* = 0) gives equal weight to species regardless of abundance and Simpson diversity (*q* = 2) gives more weight to common species (Chao et al. 2014). We also estimated species evenness as the ratio of species richness and Simpson diversity (Hill 1973; Chao et al. 2014; Morris et al. 2014).

We estimated Hill numbers using sample coverage‐based rarefaction and extrapolation with the R package *iNEXT.3d* (Chao et al. 2021, Chao and Hu 2023). To find the appropriate level of coverage to standardize samples (base coverage), we compared the maximum of observed coverage of reference samples (*n*) to the minimum of sample coverage, which is double the sample size (2*n*; Chao and Jost 2012; Chao et al. 2014). For estimating species richness, the coverage level at 2*n* is the extrapolation limit because of a large prediction bias when exceeding double the reference sample size. Our datasets have a high sample coverage and the undisturbed part of the gradient was standardized to a coverage of 98.74% for the overstorey and 91.99% for the understorey, whereas the coverage for the entire gradient including secondary forests was standardized at 98.52% for the overstorey and 83.85% for the understorey. Standardizing all datasets to the same sample completeness was not possible, because the extrapolation limit of 2*n* for species richness would have been exceeded.

### 2.4 Data Analysis

We used linear regression models to test diversity–elevation relationships and the impacts of anthropogenic disturbances at lower elevations on the shape of diversity–elevation relationships. For the undisturbed part of the gradient, we expected a monotonically decreasing pattern. Across the entire elevational gradient—including disturbed plots at low elevations—we expected the linear relationship to shift from a monotonically decreasing pattern to a hump‐shaped pattern. We therefore tested different forms of this relationship: quadratic (*x* ^2^), logarithmic (log(*x*)), and hump‐shaped (*x* ^2^ + *x*) (Irl et al. 2015).

We examined model assumptions and model fit visually using the *performance* package (Lüdecke et al. 2020). To select the most parsimonious model, we used the second‐order Akaike Information Criterion (AICc), due to the low sample size (Zuur et al. 2009). We also checked generalized least squares models with different variance structures and linear mixed‐effect models (Zuur et al. 2009), treating transect (i.e., slope aspect) as a random effect, but opted for not using them because of their higher AICc values.

We performed nonmetric multidimensional scaling (NMDS) using the *metamds* function with the *vegan* package (Oksanen et al. 2024) to examine community composition with Bray–Curtis dissimilarity for species abundance (Bray and Curtis 1957) and Jaccard dissimilarity for presence–absence data (Jaccard 1901). Differences between elevations, transects, and ecosystems were tested with a permutational multivariate analysis of variance (PERMANOVA, 999 permutations) using the *adonis2* and *pairwise.adonis* functions with the *vegan* and *pairwise.adonis* packages, respectively (Oksanen et al. 2024; Martinez Arbizu 2020). We used the R packages *dplyr* (Wickham et al. 2023) and *ggplot2* (Wickham 2016) for data manipulation and plotting, respectively, with R version 4.41 (R Core Team 2024) for all analysis and visualization.

## 3 Results

### 3.1 Observed Vascular Plant Species

We sampled a total of 17,600 m^2^ (44 plots × 400 m^2^) of overstorey vegetation and 176 m^2^ (44 plots × 4 m^2^) of understorey vegetation. We recorded a total of 12,137 individuals that were distributed among 101 species, 66 genera, and 48 families. Of the 101 species, 99 species were identified to the species level and the remaining two species were identified to the genus level. All of the identified species were native and seven species were endemic, 53 species were classified as trees, shrubs, or subshrubs and 46 species were classified as herbaceous, following Rodriguez et al. (2018). The most common families were Asteraceae (15 species), Hymenophyllaceae (8), Myrtaceae (5), Nothofagaceae (5), and Escalloniaceae (5; see species list in Table S1).

### 3.2 Estimated Species Richness

**FIGURE 3:**
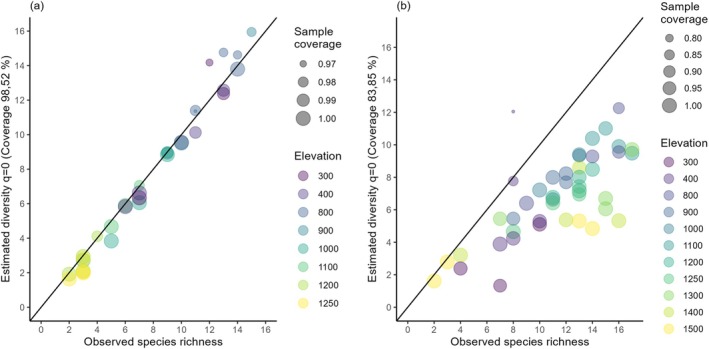
Observed species richness versus estimated species diversity (*q* = 0) of (a) overstorey and (b) understorey vegetation in temperate rainforest and alpine scrub ecosystems at Hornopirén volcano, Chile. Point size is scaled by sample coverage.

We standardized the entire overstorey dataset to a coverage level of 98.52% (respectively 98.74% for the undisturbed part of the gradient, Figure S2) and the entire understorey dataset to a coverage level of 83.85% (respectively 91.99% for the undisturbed part of the gradient, Figure S2). Standardization of estimated species richness, that is, *q* = 0 of overstorey vegetation included a mix of rarefaction and extrapolation (Figure 3a). To standardize estimated understorey richness, all assemblages had to be rarefied to the coverage level of the extrapolation to double the sample size of a plot at 400 m in Transect 2 in a secondary forest (Figure 3b). This plot showed the lowest coverage for the understorey stratum, due to its observed relative abundance distribution with a low number of individuals (*n* = 20) and a relatively high number of species (8) and singletons (5). This assemblage also showed the lowest evenness of the understorey dataset.

### 3.3 Undisturbed Elevational Gradient

**FIGURE 4:**
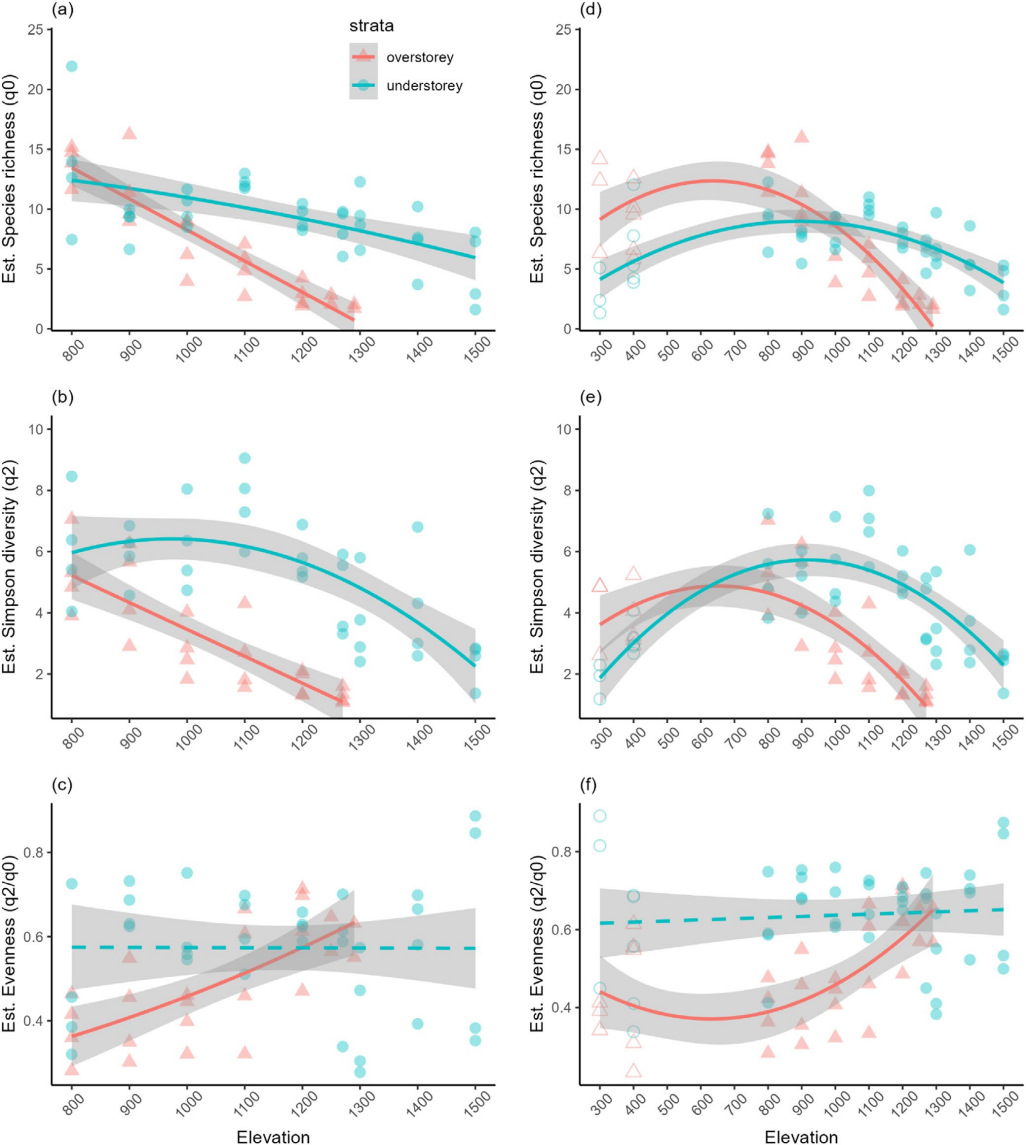
Changes in estimated species richness (*q* = 0), Simpson diversity (*q* = 2), and evenness (*q* = 2/*q* = 0) of overstorey vegetation and understorey vegetation with elevation for the undisturbed gradient (a–c) and for the entire gradient, including secondary forest (d–f) in temperate rainforest and alpine scrub ecosystems at Hornopirén volcano, Chile. Dashed lines indicate that species diversity did not vary significantly with elevation (*p* > 0.05). Gray ribbons are 95% confidence intervals. Symbols for secondary forests at 300 m and 400 m are unfilled.

For the overstorey and the understorey strata, we found monotonic decreasing relationships for estimated species richness (overstorey vegetation: *R* ^2^ = 0.83, *p* < 0.001, slope = −0.02; understorey vegetation: *R* ^2^ = 0.33, *p* < 0.001, slope = −0.009; Figure 4a), whereas understorey vegetation responded less markedly to elevation. Simpson diversity exhibited contrasting relationships with elevation across strata. For the understorey strata, Simpson diversity shifted across elevations, and exhibited a hump‐shaped pattern (understorey vegetation: *R* ^2^ = 0.45, *p* < 0.001; Figure 4b). However, Simpson diversity of overstorey vegetation showed a monotonic decreasing pattern (*R* ^2^ = 0.67, *p* < 0.001; Figure 4b). While species evenness of the understorey vegetation did not change significantly (*p* > 0.05), species evenness of the overstorey vegetation increased significantly with elevation (*R* ^2^ = 0.46, *p* < 0.001; Figure 4c).

In the alpine scrub above the treeline (1300–1500 m), we found a monotonic decreasing relationship of estimated species richness (*R* ^2^ = 0.28, *p* < 0.05), but not for Simpson diversity nor evenness. For the forested part of the gradient (800 m—treeline), we did not find a statistically significant relationship for estimated species richness, Simpson diversity, nor species evenness with elevation (*p* > 0.05). When analyzing the unstandardized data, we found that observed species richness of overstorey vegetation decreased monotonically with elevation (*R* ^2^ = 0.86, *p* < 0.001, slope = −0.02), whereas observed species richness of the understorey stratum did not respond significantly to elevation (*p* > 0.05).

### 3.4 Elevational Gradient Including Disturbed Low Elevations

For over‐ and understorey vegetation, we found that estimated as well as observed species richness showed a hump‐shaped relationship with elevation when accounting for human disturbances at low elevations (overstorey vegetation: *R* ^2^ = 0.65, *p* < 0.001; understorey vegetation: *R* ^2^ = 0.43, *p* < 0.001; Figure 4d), in contrast to the monotonically decreasing relationships when disturbed forests were excluded. Simpson diversity also exhibited a hump‐shaped relationship with elevation (overstorey: *R* ^2^ = 0.55, *p* < 0.001; understorey: *R* ^2^ = 0.57 *p* < 0.001; Figure 4e). Species evenness of overstorey vegetation was lowest in secondary forests and low elevations of old‐growth forest, and thereafter increased with elevation (*R* ^2^ = 0.36, *p* < 0.001; Figure 4f). Species evenness of the understorey vegetation did not show a statistically significant relationship with elevation (*p* > 0.05; Figure 4f).

### 3.5 Community Composition

**FIGURE 5:**
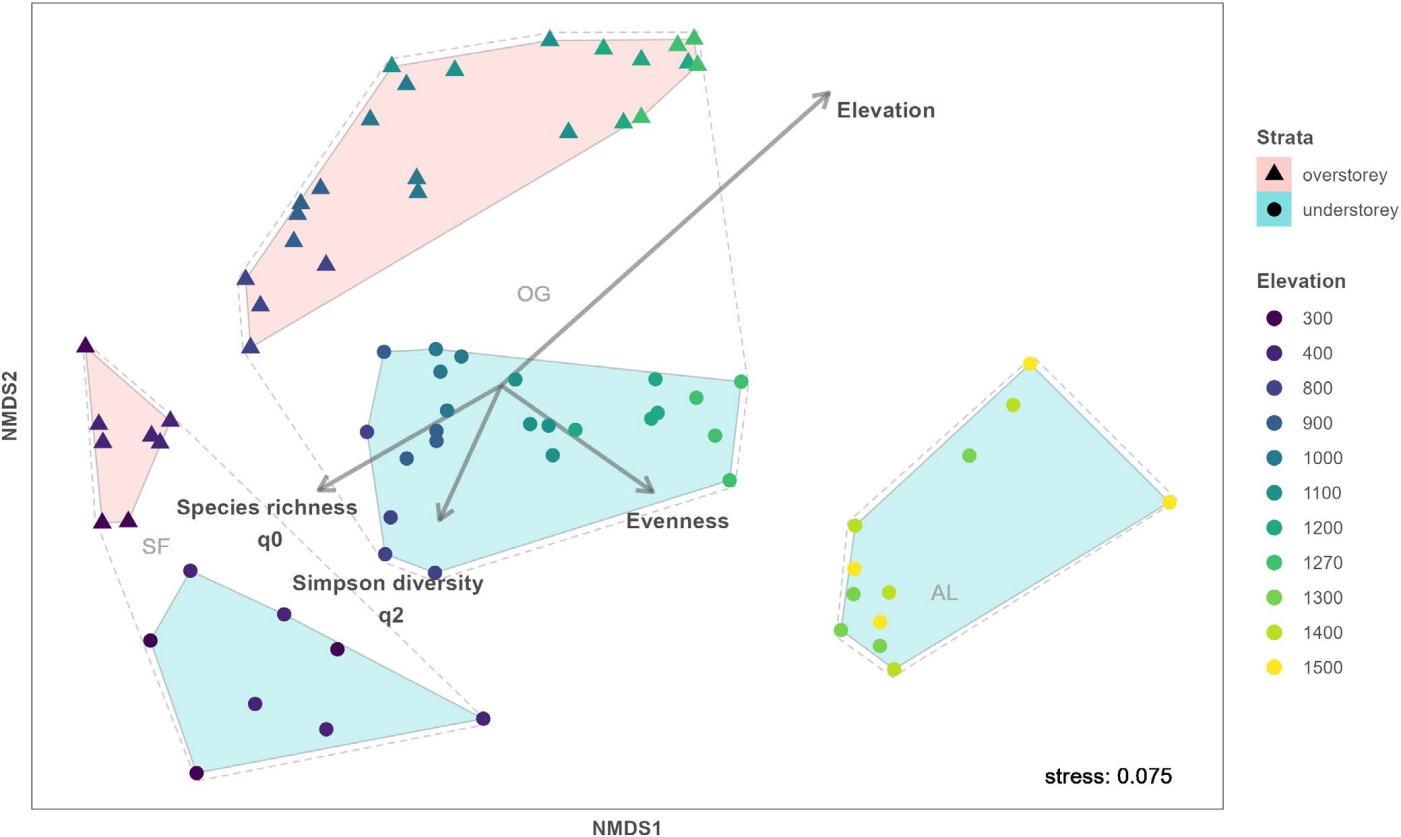
Nonmetric multidimensional scaling of species composition for overstorey strata (triangle symbols and pink convex hulls) and understorey strata (points and turquoise convex hulls) using Bray–Curtis dissimilarity across the elevational gradient in temperate rainforest and alpine scrub ecosystems at Hornopirén volcano, Chile. Transparent convex hulls with dashed lines refer to the three segments of the elevational gradient: “SF” = secondary forest (300 m and 400 m), “OG” = old growth forest (800 m–1270 m), “AL” = alpine vegetation (1300 m–1500 m).

We found that community composition varied significantly along the elevational gradient (Figure 5, Table 1, Figure S3, Table S4). Species composition also shifted along elevation for the overstorey strata (Figure S5, Table 1, Figure S6, Table S4) and the understorey strata (Figure S7, Table 1, Figure S8, Table S4). In the forested part of the gradient, species composition differed significantly between the overstorey and understorey strata (Figure 5, Table 1, Figure S3, Table S4).

Community composition differed significantly between old‐growth and secondary forests (Figure 5, Table 1, Figure S3, Table S4). Looking at each strata separately, we also found different species compositions between old‐growth and secondary forests within each strata (overstorey: Figure S5, Table 1, Figure S6, Table S4; understorey: Figure S7, Table 1, Figure S8, Table S4).

**TABLE 1:** Results of the permutational multivariate analysis of variance (PERMANOVA) evaluating the effects of elevation (Line 1–3), forest strata (Line 4), forest type (Line 5–7), and slope aspect (Line 8) on community composition of the overstorey and understorey strata in temperate rainforest and alpine scrub ecosystems at Hornopirén volcano, Chile. Dissimilarity was estimated using abundance‐based Bray–Curtis dissimilarity and 999 permutations.

	*R* ^2^	*F*	*p*
Overstorey vs. elevation	0.24	9.31	**< 0.001**
Understorey vs. elevation	0.12	5.93	**< 0.001**
Both strata vs. elevation	0.10	8.26	**< 0.001**
Overstorey vs. understorey	0.10	7.27	**< 0.001**
[Overstorey^a^] Old‐growth forest vs. secondary forest	0.20	7.48	**< 0.001**
[Understorey] Old‐growth forest vs. secondary forest	0.18	6.71	**< 0.001**
[both forest strata] Old‐growth forest vs. secondary forest	0.13	9.09	**< 0.001**
[Alpine scrub] Transect 1 vs. transect 2	0.20	2.47	**< 0.05**

*Note:* The bold values indicate the significance of the *p*‐value.

^a^ Although we found a statistically significant difference in community composition between the groups old‐growth forest and secondary forest, the assumption of homogeneity of variance within the groups was violated for the overstorey dataset, suggesting that group differences may be, at least in part, influenced by the variation in dispersion within the groups rather than the differences between the groups alone (ANOVA, *p* < 0.05).

Species composition did not differ significantly between slope aspects, neither for overstorey nor understorey strata (*p* > 0.05, Figure S9–S11). However, above the treeline, community composition varied significantly between slope aspects for Bray–Curtis dissimilarity (Table 1), but not for Jaccard dissimilarity (*p* > 0.05).

## 4 Discussion

In our study, we examined the relationship between elevation and plant diversity across forest strata along an elevational gradient in southern Chile. Our results reveal that in the undisturbed part of the elevational gradient species richness decreased monotonically, whereas species richness of overstorey vegetation decreased more markedly with elevation compared to understorey richness. Simpson diversity also decreased monotonically for overstorey vegetation, but showed a hump‐shaped relationship for understorey vegetation. Species evenness increased with elevation within the overstorey strata, but did not change significantly with elevation within understorey vegetation. Species composition differed between overstorey and understorey vegetation and shifted along elevation, respectively to forest types. As the water availability along the elevation gradient is not a limiting factor in our study area, our results therefore suggest that overstorey diversity is mainly driven by decreasing temperature with increasing elevation and that the presence of a forest canopy may buffer the effects of air temperature on understorey species diversity. Above the treeline, additional drivers of diversity besides climate, such as biotic interactions or topography, also appear to drive variation in species diversity. When extending the elevational gradient to disturbed habitats at lower elevations, our analyses revealed lower species richness and Simpson diversity in secondary forests compared to old‐growth forests at higher elevations, which led to a change in the shape of diversity–elevation relationships, from monotonically decreased to hump‐shaped. Furthermore, we found differences in community composition between secondary and old‐growth forests. Our results therefore highlight how human activity is reshaping biodiversity patterns in mountain ecosystems in southern South America.

### 4.1 Diversity–Elevation Relationships Across Forest Strata in Undisturbed Habitats

According to our hypothesis, we found that estimated and observed species richness decreased monotonically with elevation for overstorey vegetation (Figure 4a). The marginal differences between the model parameters for estimated and observed species richness likely reflect the high sample coverage of our overstorey dataset (Figure 3a, Figure S2a). Due to the dependence of plants on spatial variation in water and energy, climatic constraints have been proposed to be the most important factors determining vascular plant richness patterns at broad spatial scales (O'Brien 1993; Francis and Currie 2003; Currie et al. 2004; Barthlott et al. 2005; Kreft and Jetz 2007; Zhang et al. 2014). Decreasing temperature with increasing elevation has been identified as the principal limiting factor for vascular plants (Peters et al. 2016), especially for trees (e.g., Leathwick et al. 1998; Sharma et al. 2019; Monge‐González et al. 2020). This is likely the case for the present study, as it is unlikely that water availability is limiting at any point along the elevation gradient, given the high precipitation in the study area (3000–5000 mm yr). According to the ambient energy hypothesis, decreasing temperature with elevation acts as a strong environmental filter, operating on species without an evolved frost tolerance (Hawkins et al. 2003; Evans et al. 2005; Sommer et al. 2010; Zanne et al. 2014). Most vascular plants have evolved under wet, tropical conditions (Crane and Lidgard 1989; Wing and Boucher 1998), and the ancestral lineages of the study area evolved in a significantly warmer climate during the Paleogene (Aizen and Ezcurra 2008; Jaramillo and Cárdenas 2013; Segovia and Armesto 2015). Thus, the interplay between the tendency of lineages to maintain their ancestral niches (niche conservatism, Wiens and Donoghue 2004) and to adapt physiologically to freezing conditions jointly determines species distributions, particularly along elevational gradients (Zanne et al. 2014). In support of this idea, we found that overstorey species richness was higher at lower elevations, where warmer temperatures allow species with and without frost tolerance to co‐occur. In line with our results, surveys of primary forests in New Zealand, which have climatic and floristic similarities to the study area (McGlone et al. 2016), reported monotonic decreases in tree species richness with elevation (Ogden 1995) and a strong correlation of species richness with mean annual temperature (MAT; Leathwick et al. 1998). MAT was also identified as being the best predictor for tree species richness in temperate forests of south‐eastern Australia (Austin et al. 1996).

The monotonic decrease in species richness and Simpson diversity of overstorey vegetation along the elevation gradient reveals that the high tree and shrub species richness at low elevations was largely driven by rare species, which led to low evenness (Figure 4b,c). Moreover, the slopes of the modeled species richness–elevation and Simpson–diversity relationships indicate that many of the shade‐tolerant evergreen trees and shrubs or conifers (e.g., *Amomyrtus luma*, *Luma apiculata* , both Myrtaceae; *Podocarpus nubigena*) were already less abundant due to their elevational limits (Rodriguez et al. 2018), before being entirely filtered out at higher elevations. Thus, the likely impacts of environmental filtering on overstorey communities were also manifested via their abundance distributions. The combination of the decreasing abundance of shade‐tolerant, low‐elevation specialists and the increasing dominance of *Nothofagus* spp., due to reduced competition and less dependence on coarse‐scale disturbances than at lowest elevations (Veblen et al. 1996; Pollmann and Veblen 2004), led to a high overall overstorey richness and uneven abundance distributions. The increase in evenness toward the treeline is apparently connected to the strong decrease in tree and shrub species richness, with only *Nothofagus pumilio, N. antarctica* and *Escallonia alpina* (Escalloniaceae) remaining at the highest elevations.

In contrast to the overstorey dataset, we found striking differences in the diversity–elevation relationship between estimated and observed species richness for the understorey stratum. Whereas estimated species richness of the understorey stratum decreased monotonically with elevation (Figure 4a), observed species richness did not change significantly along elevation. These findings mirror the lower sample coverage of the understorey dataset (Figure 3b, Figure S2b) and underpin the necessity of data standardization to facilitate a fair comparison between samples of different completeness (Chao and Jost 2012).

Our findings that estimated understorey species richness changed less markedly with elevation than estimated overstorey species richness (slope overstorey stratum = −0.02; slope understorey stratum = −0.009; Figure 4a) and did not significantly decrease along elevation in the forested part of the gradient, was in line with our hypothesis and supports the idea that the impact of environmental filtering varies between forest strata (Körner 2007; Zellweger et al. 2020). The temperature and its effects on diversity differ across forest strata due to plant size, growth form and distance from the ground. The air temperature that overstorey vegetation is exposed to varies significantly from temperatures near the ground surface or under vegetation cover, which is likely more relevant for understorey vegetation (Lembrechts et al. 2021). Thus, overstorey vegetation buffers understorey vegetation from extreme temperatures, resulting in diversity patterns that are more decoupled from temperature variation (Körner 2007). Hence, it is likely that local factors besides climate, such as soil temperature and properties, light availability, disturbance, or competition drive understorey patterns in forests (Quigley and Platt 2003; Wang et al. 2009; Zhang et al. 2014). The monotonic decreases in estimated tree and shrub richness and a less marked response of estimated understorey vegetation to decreasing temperature with elevation that we observed are consistent with studies from other temperate regions, such as New Zealand and temperate north‐east China (Ohlemüller and Wilson 2000; Wang et al. 2009), suggesting that nonclimatic drivers shape diversity patterns of understorey vegetation.

Across the elevation gradient, we found hump‐shaped patterns of Simpson diversity for understorey vegetation (Figure 4b). These patterns suggest that higher proportions of rare species occur at the highest and lowest elevations. Our result supports the findings of studies in Spain, where endemicity was highest toward the base and the top of the elevational gradient and rare species were concentrated at higher elevations (Irl et al. 2015; Irl 2016). In old‐growth forests, the large proportion of rare species within understorey vegetation was linked to the high tree and shrub species richness. The skewed abundance distributions can be attributed to the lower abundances of woody regeneration (in understorey strata), compared to the dominant herbaceous understorey (e.g., *Asteranthera ovata* , Gesneriaceae, or the ferns such as *Hymenophyllum* spp., Hymenophyllaceae; *Sticherus quadripartitus*, Gleicheniaceae; *Blechnum magellanicum*, Blechnaceae). Our results suggest that in old‐growth forest, where environmental stress is low due to moderate temperatures and high water availability, the tolerance–fecundity tradeoff (Muller‐Landau 2010) favors the regeneration of herbaceous understorey species due to their smaller seed size (respectively fern‐spores), compared with the overstorey plant species that tend to have larger seed sizes. Thus, our approach to investigate patterns of diversity–elevation relationships separately per stratum enabled us to disentangle how community structure shifts along elevation gradients and to deconstruct which forest strata are more sensitive to the particular diversity metric.

In the alpine scrub, we found the largest variation in species richness between plots at the same elevations along the gradient, apart from the assemblages at 800 m (Figure 4a). Furthermore, species composition varied significantly between slope aspects in the alpine scrub, in contrast to old‐growth forests, where it did not differ between slope aspects (Figure 5). Thus, our hypothesis was partly rejected. Most likely, in the forested part of the gradient, potential climatic differences between the slope aspects were mediated by the overstorey layers that created homogenous abiotic conditions per elevational band, thus enabling a similar species richness and composition. In contrast, low‐stature vegetation in the alpine scrub is generally more directly exposed to the harsher climatic conditions due to the absence of buffering effects of overstorey vegetation and may respond more strongly to temperature (Bergstedt and Milberg 2001; Mestre et al. 2017). Above the treeline, our results likely reflect a response to more heterogeneous conditions than to temperature, as species richness and community composition did not vary linearly with elevation. Therefore, our results support the idea that above the treeline, where environmental stress increases due to the absence of overstorey vegetation, local factors that create an array of microhabitat conditions, such as slope aspect (i.e., insolation, wind and rain exposure), small‐scale topography, steepness, and biotic interactions, become increasingly important in determining spatial patterns of species diversity and community composition (Wang et al. 2009; López‐Angulo et al. 2018; Ezcurra and Gavini 2020).

According to the stress–gradient–hypothesis (SGH), facilitative interactions are expected to be prevalent in stressful environments and to enhance species richness (Bertness and Callaway 1994; Duarte et al. 2020). Nursing effects of cushion plants and dwarf shrubs are known to play an important facilitative role in Andean alpine environments (Badano and Cavieres 2006; Cavieres and Badano 2009; Cavieres et al. 2016; Ezcurra and Gavini 2020). Although we did not measure biotic interactions, it is likely that cushion‐growth plants and dwarf shrubs such as *Azorella ranunculus* (Apiaceae), *Tribeles australis*, or *Empetrum rubrum* created microhabitats for the abundant rosette plant species like *Valeriana fonckii* (Valerianaceae), *Perezia pedicularidifolia* (Asteraceae) or *Senecio* spp. by attenuating extreme temperatures, buffering diurnal changes in temperature, protecting other plants from strong winds, or possibly providing nutrients (Piper et al. 2019). The hump‐shaped patterns of Simpson diversity for understorey vegetation (Figure 4b) provide evidence in support of this idea, as asymmetric abundance distributions above the treeline capture the co‐occurrence of highly abundant rosette plants and less abundant cushion plants and dwarf shrubs. Whereas SGH primarily refers to species richness, here we propose that the observed abundance distributions and the differences in community composition between slope aspects in alpine ecosystems may indicate the potential importance of facilitative interactions in stressful environments. However, further field and experimental studies would be needed to confirm that skewed abundance distributions are determined by facilitative interactions in alpine or similarly stressful ecosystems, such as arid environments and drylands (He et al. 2013; Eibes et al. 2021; Mandakovic et al. 2023).

### 4.2 Disturbances May Alter Diversity–Elevation Relationships

With the exception of Simpson diversity of understorey vegetation, we found that including disturbed sites at lower elevations changed the shape of the diversity–elevation relationship, from monotonically decreasing to hump‐shaped (Figure 4), supporting our hypothesis (Figure 1b). Analyzing observed species richness did not lead to a different diversity–elevation relationship than that when using estimated species richness, as it also showed a hump‐shaped pattern for both strata (Figure 4d, Figure S2). While secondary forests were highly variable in terms of species diversity and composition, likely reflecting varying impacts of the observed former and ongoing land uses, for example, selective wood extraction, cattle grazing or logging, their species richness was lower than one would expect based on the predictions of the models fitted from the undisturbed portion of the elevation gradient (Figure 1b).

Our results are consistent with global studies (Murphy and Romanuk 2014; Newbold et al. 2015) and a study from Mt. Kilimanjaro, which showed that land use at lower elevations shifted the diversity–elevation relationship from monotonically decreasing to hump‐shaped and altered species composition (Peters et al. 2019). This suggests that comparing expected diversity–elevation relationships for undisturbed habitats with observed diversity–elevation relationships from disturbed and undisturbed habitats might be a viable alternative for detecting the effects of human activities on diversity–elevation relationships when undisturbed habitats no longer exist, given (i) the prevalence of high land‐use intensity at lower elevations (Nogués‐Bravo et al. 2008; McCain and Grytnes 2010; Costa et al. 2023), and (ii) the increasing scarcity of forests with high ecosystem integrity (Grantham et al. 2020). It is crucial to mention that our conclusions about the form of the diversity–elevation relationship are applicable for humid regions with low energy input, where decreasing temperature with elevation is most likely to constrain diversity, instead of water availability, which is abundant along the gradient and at low elevations (Hawkins et al. 2003; Kreft and Jetz 2007; Grytnes and McCain 2013).

Interestingly, a meta‐study of Costa et al. (2023) on biodiversity and elevational gradients did not find any effects of disturbances on the species richness–elevation relationship. However, they assumed that no disturbances occurred within a legally protected area. Given the observed disturbance at low elevations in the study area (approx. < 800 m), that is, inside and outside of Hornopirén National Park's borders and that globally only just more than half of the forests inside protected areas have high landscape‐level integrity (Grantham et al. 2020), their findings merit consideration (also see Werenkraut and Ruggiero 2011). Rather, our results provide empirical evidence that anthropogenic disturbances appear to have altered local biodiversity within Hornopirén National Park as well as in adjacent areas beyond its administrative borders. Our findings thus shed light on human induced threats to biodiversity which is pivotal for the conservation of forest ecosystems in the Chilean Patagonia (Pliscoff et al. 2023). Moreover, these findings underpin the importance of monitoring and preserving biodiversity inside and outside of Chilean Patagonia National Parks (Arroyo 2023) and conservation areas in southern South America.

In agreement with our findings, previous studies in southern South America, have found that the co‐occurrence of human disturbances and cattle grazing has been found to negatively influence diversity in *Nothofagus* forests by halting tree regeneration, reducing the species diversity of understorey vegetation, and decreasing species evenness (Raffaele et al. 2007; Vila and Borrelli 2011; Piazza et al. 2016; Soto et al. 2019). Also, biodiversity declines due to human activity have been extensively documented for southern Chile previously, but usually at larger spatial scales, and not along elevational gradients (Smith‐Ramírez 2004; Armesto et al. 2009; Miranda et al. 2017; Rodríguez‐Echeverry et al. 2018). Our study therefore adds to this body of literature by showing that human activity is likely altering the ecological processes that give rise to diversity–elevation relationships.

As expected according to our hypothesis, we found that species composition was significantly different in disturbed secondary forests compared to old‐growth forests (Figure 5). Our results support those of previous studies that the fast proliferation of bamboos (*Chusquea* spp.) in canopy openings following anthropogenic disturbances often impedes the regeneration of shade‐intolerant or semi‐tolerant species that are typical of old‐growth forests such as *Nothofagus* spp. (Donoso 1998; González et al. 2002; Holz and Veblen 2006), favoring the advance regeneration of shade‐tolerant tree species such as *Laureolopsis philippiana* (Atherospermataceae), *Archdasyphyllum diacanthoides* (Asteraceae), *Amomyrtus luma*, *Luma apiculata* , and *Myrceugenia planipes* (Myrtaceae) (Veblen et al. 1996; Donoso et al. 1999; González et al. 2002; Bannister and Donoso 2013), or typical pioneer species such as *Drimys winteri* (Winteraceae), (Navarro et al. 1999; Donoso et al. 2007; Gutiérrez et al. 2004; Soto et al. 2019). Thus, our study reflects the legacy of different former and ongoing land uses in Patagonian temperate forests (Joelson et al. 2025), but additionally shows that human activity may also lead to biotic differentiation and not—as expected—biotic homogenization (Blowes et al. 2024).

## 5 Conclusion

Our study shows that diversity–elevation relationships depend on forest strata, highlighting that deviations from expected changes in diversity along elevation gradients may reflect shifts in community structure and the relative importance of specific drivers, such as climate or biotic interactions. Furthermore, at disturbed low elevations our results point towards land use as a cause of altered diversity–elevation relationships and species composition of vascular plants in austral temperate ecosystems. Our results underscore the critical importance of monitoring biodiversity both inside and outside protected areas to detect potentially significant changes in local biodiversity patterns associated with human activities. Besides the fundamental importance of investigating biodiversity patterns and their underlying drivers in mountain ecosystems, we argue that sustainable management and conservation strategies are essential for mitigating further human impacts on these unique ecosystems regardless of their conservation status. Further biodiversity monitoring initiatives should be encouraged to expand the traditional set of biodiversity measures, i.e., species richness, to include biodiversity facets such as phylogenetic and functional diversity, hopefully leading to a more holistic understanding of processes generating diversity patterns in temperate South America—a globally recognized biodiversity hotspot under increasing anthropogenic pressure that is underrepresented in biodiversity research.

## Author Contributions

**T. Michelt:** conceptualization (lead), data curation (lead), formal analysis (lead), funding acquisition (lead), investigation (lead), methodology (lead), project administration (lead), resources (lead), software (lead), visualization (lead), writing – original draft (lead). **D. Craven:** conceptualization (equal), data curation (equal), formal analysis (equal), funding acquisition (equal), methodology (equal), supervision (equal), writing – review and editing (equal). **V. Peréz‐Tello:** data curation (supporting), investigation (supporting), writing – original draft (supporting). **S. D. H. Irl:** conceptualization (equal), formal analysis (equal), funding acquisition (equal), methodology (equal), project administration (equal), supervision (equal), writing – review and editing (equal).

## Conflicts of Interest

The authors declare no conflicts of interest.

## Supporting information


**Appendix S1:** ece372515‐sup‐0001‐AppendixS1.zip.

## Data Availability

All data is available at the DRYAD repository: https://doi.org/10.5061/dryad.6wwpzgnc6.

## References

[ece372515-bib-0001] Aizen, M. A. , and C. Ezcurra . 2008. “Do Leaf Margins of the Temperate Forest Flora of Southern South America Reflect a Warmer Past?” Global Ecology and Biogeography 17: 164–174.

[ece372515-bib-0002] Allen, A. P. , J. H. Brown , and J. F. Gillooly . 2002. “Global Biodiversity, Biochemical Kinetics, and the Energetic‐Equivalence Rule.” Science 297: 1545–1548. 12202828 10.1126/science.1072380

[ece372515-bib-0003] Antonelli, A. , W. D. Kissling , S. G. A. Flantua , et al. 2018. “Geological and Climatic Influences on Mountain Biodiversity.” Nature Geoscience 11: 718–725.

[ece372515-bib-0004] Armesto, J. J. , P. Leon‐Lobos , and M. T. K. Arroyo . 1997. “Los bosques templados del sur de Chile y Argentina una isla biogeografica.” In Ecologia de los bosques nativos de Chile, edited by J. J. Armesto , C. Villagran , and M. T. K. Arroyo . Editorial Universitaria.

[ece372515-bib-0005] Armesto, J. J. , C. Smith‐Ramírez , M. R. Carmona , et al. 2009. “Old‐Growth Temperate Rainforests of South America: Conservation, Plant–Animal Interactions, and Baseline Biogeochemical Processes.” In Old‐Growth Forests, edited by C. Wirth , G. Gleixner , and M. Heimann . Springer.

[ece372515-bib-0006] Arroyo, M. T. K. 2023. “A Brief Vision of the Past, Present, and Future of Chilean Patagonia.” In Conservation in Chilean Patagonia—Assessing the State of Knowledge, Opportunities, and Challenges, edited by J. C. Castilla , J. J. Armesto , M. J. Martínez‐Harms , and D. Tecklin . Pontificia Universidad Católica de Chile.

[ece372515-bib-0007] Arroyo, M. T. K. , I. Tamburrino , V. Robles , K. Robles , and L. V. Morales . 2024. “Data on Vascular Plant Species Composition Along Two Elevation Gradients in the High Andes of Central Chile (33° S).” Data in Brief 57: 111128. 39640396 10.1016/j.dib.2024.111128 PMC11617968

[ece372515-bib-0008] Austin, M. P. , J. G. Pausas , and A. O. Nicholls . 1996. “Patterns of Tree Species Richness in Relation to Environment in Southeastern New South Wales, Australia.” Austral Ecology 21: 154–164.

[ece372515-bib-0009] Badano, E. I. , and L. A. Cavieres . 2006. “Impacts of Ecosystem Engineers on Community Attributes: Effects of Cushion Plants at Different Elevations of the Chilean Andes.” Diversity and Distributions 12: 388–396.

[ece372515-bib-0010] Bannister, J. , and P. Donoso . 2013. “Forest Typification to Characterize the Structure and Composition of Old‐Growth Evergreen Forests on Chiloe Island, North Patagonia (Chile).” Forests 4: 1087–1105.

[ece372515-bib-0011] Barthlott, W. , J. Mutke , D. Rafiqpoor , G. Kier , and H. Kreft . 2005. “Global Centers of Vascular Plant Diversity.” Nova Acta Leopoldina 92: 61–83.

[ece372515-bib-0012] Becerra, P. I. 2016. “Relationship Between Climate and Geographical Variation of Local Woody Species Richness Within the Mediterranean‐Type Region of Chile.” Revista Chilena de Historia Natural 89: 1–11.

[ece372515-bib-0013] Bergstedt, J. , and P. Milberg . 2001. “The Impact of Logging Intensity on Field‐Layer Vegetation in Swedish Boreal Forests.” Forest Ecology and Management 154: 105–115.

[ece372515-bib-0014] Bertness, M. D. , and R. Callaway . 1994. “Positive Interactions in Communities.” Trends in Ecology & Evolution 9: 191–193. 21236818 10.1016/0169-5347(94)90088-4

[ece372515-bib-0015] Bhatta, K. P. , B. A. Robson , M. K. Suwal , and O. R. Vetaas . 2021. “A Pan‐Himalayan Test of Predictions on Plant Species Richness Based on Primary Production and Water‐Energy Dynamics.” Frontiers of Biogeography 13, no. 3: e49459.

[ece372515-bib-0016] Blowes, S. A. , B. McGill , V. Brambilla , et al. 2024. “Synthesis Reveals Approximately Balanced Biotic Differentiation and Homogenization.” Science Advances 10: 1–10. 10.1126/sciadv.adj9395 PMC10881054 38381832

[ece372515-bib-0017] Bonpland, A. , and A. von Humboldt . 1807. Ideen zu einer Geographie der Pflanzen nebst einem Naturgemälde der Tropenländer. Cotta.

[ece372515-bib-0018] Boyle, B. , N. Hopkins , Z. Lu , et al. 2013. “The Taxonomic Name Resolution Service: An Online Tool for Automated Standardization of Plant Names.” BMC Bioinformatics 14: 1–14. 23324024 10.1186/1471-2105-14-16 PMC3554605

[ece372515-bib-0019] Bray, J. R. , and J. T. Curtis . 1957. “An Ordination of the Upland Forest Communities of Southern Wisconsin.” Ecological Monographs 27: 325–349.

[ece372515-bib-0020] Cavieres, L. A. , and E. I. Badano . 2009. “Do Facilitative Interactions Increase Species Richness at the Entire Community Level?” Journal of Ecology 97: 1181–1191.

[ece372515-bib-0021] Cavieres, L. A. , C. Hernández‐Fuentes , A. Sierra‐Almeida , and Z. Kikvidze . 2016. “Facilitation Among Plants as an Insurance Policy for Diversity in Alpine Communities.” Functional Ecology 30: 52–59.

[ece372515-bib-0022] Chao, A. , N. J. Gotelli , T. C. Hsieh , et al. 2014. “Rarefaction and Extrapolation With Hill Numbers: A Framework for Sampling and Estimation in Species Diversity Studies.” Ecological Monographs 84: 45–67.

[ece372515-bib-0023] Chao, A. , P. A. Henderson , C.‐H. Chiu , et al. 2021. “Measuring Temporal Change in Alpha Diversity: A Framework Integrating Taxonomic, Phylogenetic and Functional Diversity and the iNEXT.3D Standardization.” Methods in Ecology and Evolution 12: 1926–1940.

[ece372515-bib-0024] Chao, A. , and K.‐H. Hu . 2023. “The iNEXT.” 3D Package: Interpolation and Extrapolation for Three Dimensions of Biodiversity. R Package Available From CRAN.

[ece372515-bib-0025] Chao, A. , and L. Jost . 2012. “Coverage‐Based Rarefaction and Extrapolation: Standardizing Samples by Completeness Rather Than Size.” Ecology 93: 2533–2547. 23431585 10.1890/11-1952.1

[ece372515-bib-0026] Chape, S. , ed. 2008. The World's Protected Areas. Status, Values and Prospects in the 21st Century. Univ. of California Pr.

[ece372515-bib-0028] CONAF . 1999. “Plan de Manejo Parque Nacional Hornopirén.”

[ece372515-bib-0029] Costa, F. V. d. , A. B. Viana‐Júnior , R. Aguilar , F. A. O. Silveira , and T. Cornelissen . 2023. “Biodiversity and Elevation Gradients: Insights on Sampling Biases Across Worldwide Mountains.” Journal of Biogeography 50, no. 11: 1879–1889.

[ece372515-bib-0030] Crane, P. R. , and S. Lidgard . 1989. “Angiosperm Diversification and Paleolatitudinal Gradients in Cretaceous Floristic Diversity.” Science 246: 675–678. 17833420 10.1126/science.246.4930.675

[ece372515-bib-0031] Currie, D. J. , G. G. Mittelbach , H. V. Cornell , et al. 2004. “Predictions and Tests of Climate‐Based Hypotheses of Broad‐Scale Variation in Taxonomic Richness.” Ecology Letters 7: 1121–1134.

[ece372515-bib-0034] Donoso, C. 1981. Tipos forestales de los bosques nativos de Chile. Proyecto CONAF/PNUD/FAO.

[ece372515-bib-0032] Donoso, C. 1998. Bosques templados de Chile y Argentina. Variación, estructura y dinámica. 4th ed. Ed. Universitaria.

[ece372515-bib-0033] Donoso, C. , P. Donoso , M. González , and V. Sandoval . 1999. “Los bosques siempreverdes.” In Silvicultura de los bosques nativos de Chile, edited by C. Donoso and A. L. Aguilar . Ed. Universitaria Univ.

[ece372515-bib-0035] Donoso, P. J. , D. P. Soto , and R. A. Bertín . 2007. “Size–Density Relationships in *Drimys winteri* Secondary Forests of the Chiloe Island, Chile: Effects of Physiography and Species Composition.” Forest Ecology and Management 239: 120–127.

[ece372515-bib-0037] Duarte, M. , M. Verdú , L. A. Cavieres , and R. O. Bustamante . 2020. “Plant–Plant Facilitation Increases With Reduced Phylogenetic Relatedness Along an Elevation Gradient.” Oikos 130: 248–259.

[ece372515-bib-0038] Echeverría, C. , A. C. Newton , A. Lara , J. M. R. Benayas , and D. A. Coomes . 2007. “Impacts of Forest Fragmentation on Species Composition and Forest Structure in the Temperate Landscape of Southern Chile.” Global Ecology and Biogeography 16: 426–439.

[ece372515-bib-0039] Eibes, P. M. , J. Eisenbacher , C. Beierkuhnlein , et al. 2021. “Co‐Occurrence Frequency in Vegetation Patches Decreases Towards the Harsh Edge Along an Arid Volcanic Elevational Gradient.” Frontiers of Biogeography 13, no. 3: e49743.

[ece372515-bib-0040] Elsen, P. R. , W. B. Monahan , and A. M. Merenlender . 2018. “Global Patterns of Protection of Elevational Gradients in Mountain Ranges.” Proceedings of the National Academy of Sciences of the United States of America 115: 6004–6009. 29784825 10.1073/pnas.1720141115 PMC6003333

[ece372515-bib-0041] Elsen, P. R. , W. B. Monahan , and A. M. Merenlender . 2020. “Topography and Human Pressure in Mountain Ranges Alter Expected Species Responses to Climate Change.” Nature Communications 11: 1–10. 10.1038/s41467-020-15881-x PMC7181879 32332913

[ece372515-bib-0042] Evans, K. L. , P. H. Warren , and K. J. Gaston . 2005. “Species‐Energy Relationships at the Macroecological Scale: A Review of the Mechanisms.” Biological Reviews of the Cambridge Philosophical Society 80: 1–25. 15727036 10.1017/s1464793104006517

[ece372515-bib-0043] Ezcurra, C. , and S. S. Gavini . 2020. “Alpine Plant Diversity in Temperate Mountains of South America.” In Encyclopedia of the World's Biomes, edited by M. I. Goldstein and D. A. DellaSala , 323–334. Elsevier.

[ece372515-bib-0044] Farwig, N. , N. Sajita , G. Schaab , and K. Böhning‐Gaese . 2008. “Human Impact Diminishes Seedling Species Richness in Kakamega Forest, Kenya.” Basic and Applied Ecology 9: 383–391.

[ece372515-bib-0045] Ferreyra, M. , A. Cingolani , C. Ezcurra , and D. Bran . 1998. “High‐Andean Vegetation and Environmental Gradients in Northwestern Patagonia, Argentina.” Journal of Vegetation Science 9: 307–316.

[ece372515-bib-0046] Floyd, M. L. , T. L. Fleischner , D. Hanna , and P. Whitefield . 2003. “Effects of Historic Livestock Grazing on Vegetation at Chaco Culture National Historic Park, New Mexico.” Conservation Biology 17: 1703–1711.

[ece372515-bib-0047] Francis, A. P. , and D. J. Currie . 2003. “A Globally Consistent Richness‐Climate Relationship for Angiosperms.” American Naturalist 161: 523–536. 10.1086/368223 12776882

[ece372515-bib-0048] Gaston, K. J. 2000. “Global Patterns in Biodiversity.” Nature 405: 220–227. 10821282 10.1038/35012228

[ece372515-bib-0049] Geiger, G. 1961. “Köppen‐Geiger/Klima der Erde.” Überarbeitete Neuausgabe von Geiger, G. Klett‐Perthes Gotha.

[ece372515-bib-0050] Global Volcanism Program . 2013. Hornopirén. Volcanoes of the World, edited by E. Venzke . Distributed by Smithsonian Institution.

[ece372515-bib-0051] González, M. E. , T. T. Veblen , C. Donoso , and L. Valeria . 2002. “Tree Regeneration Responses in a Lowland Nothofagus‐Dominated Forest After Bamboo Dieback in South‐Central Chile.” Plant Ecology 161: 59–73.

[ece372515-bib-0052] Gotelli, N. J. , and R. Colwell . 2011. “Estimating Species Richness.” In Biological Diversity: Frontiers in Measurement and Assessment, edited by A. E. Magurran and B. J. McGill . Oxford Univ. Press; Oxford University Press.

[ece372515-bib-0053] Grantham, H. S. , A. Duncan , and T. D. Evans . 2020. “Anthropogenic Modification of Forests Means Only 40% of Remaining Forests Have High Ecosystem Integrity.” Nature Communications 11: 5978. 10.1038/s41467-020-19493-3 PMC7723057 33293507

[ece372515-bib-0054] Grytnes, J. A. , E. Heegaard , and P. G. Ihlen . 2006. “Species Richness of Vascular Plants, Bryophytes, and Lichens Along an Altitudinal Gradient in Western Norway.” Acta Oecologica 29: 241–246.

[ece372515-bib-0055] Grytnes, J.‐A. , and C. M. McCain . 2013. “Elevational Trends in Biodiversity.” In Encyclopedia of Biodiversity, edited by S. A. Levin . Academic Press.

[ece372515-bib-0056] Guo, Q. , D. A. Kelt , Z. Sun , et al. 2013. “Global Variation in Elevational Diversity Patterns.” Scientific Reports 3: 1–7. 10.1038/srep03007 PMC6505670 24157658

[ece372515-bib-0058] Gutiérrez, A. G. , J. J. Armesto , and J. C. Aravena . 2004. “Disturbance and Regeneration Dynamics of an Old‐Growth North Patagonian Rain Forest in Chiloé Island, Chile.” Journal of Ecology 92: 598–608.

[ece372515-bib-0057] Gutiérrez, A. G. , J. J. Armesto , J.‐C. Aravena , et al. 2009. “Structural and Environmental Characterization of Old‐Growth Temperate Rainforests of Northern Chiloé Island, Chile: Regional and Global Relevance.” Forest Ecology and Management 258: 376–388.

[ece372515-bib-0059] Hawkins, B. A. , R. Field , H. V. Cornell , et al. 2003. “Energy, Water, and Broad‐Scale Geographic Patterns of Species Richness.” Ecology 84: 3105–3117.

[ece372515-bib-0060] He, Q. , M. D. Bertness , and A. H. Altieri . 2013. “Global Shifts Towards Positive Species Interactions With Increasing Environmental Stress.” Ecology Letters 16, no. 5: 695–706. 23363430 10.1111/ele.12080

[ece372515-bib-0061] Hill, M. O. 1973. “Diversity and Evenness: A Unifying Notation and Its Consequences.” Ecology 54: 427–432.

[ece372515-bib-0063] Hobbs, R. J. 2001. “Synergisms Among Habitat Fragmentation, Livestock Grazing, and Biotic Invasions in Southwestern Australia.” Conservation Biology 15: 1522–1528.

[ece372515-bib-0064] Holz, C. A. , and T. T. Veblen . 2006. “Tree Regeneration Responses to *Chusquea montana* Bamboo Die‐Off in a Subalpine Nothofagus Forest in the Southern Andes.” Journal of Vegetation Science 2006: 19–28.

[ece372515-bib-0066] iPlant Collaborative . 2020. “The Taxonomic Name Resolution Service.” https://tnrs.iplantcollaborative.org.

[ece372515-bib-0067] Irl, S. D. H. , D. E. V. Harter , M. J. Steinbauer , et al. 2015. “Climate vs. Topography–Spatial Patterns of Plant Species Diversity and Endemism on a High‐Elevation Island.” Journal of Ecology 103: 1621–1633.

[ece372515-bib-0068] Irl, S. D. H. 2016. “Plant Diversity on High Elevation Islands—Drivers of Species Richness and Endemism.” Frontiers of Biogeography 8: 1–8.

[ece372515-bib-0069] Jaccard, P. 1901. “Étude Comparative de la Distribution florale dans une portion des Alpes et du Jura.” Bulletin de la Societe Vaudoise des Sciences Naturelles 37: 547–579.

[ece372515-bib-0070] Jaramillo, C. , and A. Cárdenas . 2013. “Global Warming and Neotropical Rainforests: A Historical Perspective.” Annual Review of Earth and Planetary Sciences 41: 741–766.

[ece372515-bib-0071] Joelson, N. Z. , E. Schneider , S. Heinrichs , et al. 2025. “A 50‐Year Perspective on Conservation Challenges and Legacy Effects in Temperate Patagonian Forests.” Biological Conservation 306: 111124.

[ece372515-bib-0072] Karger, D. N. , O. Conrad , J. Böhner , et al. 2018. “Climatologies at High Resolution for the Earth's Land Surface Areas.” EnviDat. 10.1038/sdata.2017.122 PMC5584396 28872642

[ece372515-bib-0077] Körner, C. 2000. “Why Are There Global Gradients in Species Richness? Mountains Might Hold the Answer.” Trends in Ecology & Evolution 15: 513–514.

[ece372515-bib-0074] Körner, C. 2004. “Mountain Biodiversity, Its Causes and Function.” Ambio: A Journal of the Human Environment 33: 11. 15575177

[ece372515-bib-0076] Körner, C. 2007. “The Use of ‘Altitude’ in Ecological Research.” Trends in Ecology & Evolution 22: 569–574. 17988759 10.1016/j.tree.2007.09.006

[ece372515-bib-0073] Körner, C. , W. Jetz , J. Paulsen , et al. 2017. “A Global Inventory of Mountains for Bio‐Geographical Applications.” Alpine Botany 127: 1–15.

[ece372515-bib-0075] Körner, C. , and E. Spehn . 2019. “A Humboldtian View of Mountains.” Science 365: 1061. 31515359 10.1126/science.aaz4161

[ece372515-bib-0078] Kreft, H. , and W. Jetz . 2007. “Global Patterns and Determinants of Vascular Plant Diversity.” Proceedings of the National Academy of Sciences of the United States of America 104: 5925–5930. 17379667 10.1073/pnas.0608361104 PMC1851593

[ece372515-bib-0079] Leathwick, J. R. , B. R. Burns , and B. D. Clarkson . 1998. “Environmental Correlates of Tree Alpha‐Diversity in New Zealand Primary Forests.” Ecography 21: 235–246.

[ece372515-bib-0080] Lembrechts, J. J. , J. van den Hoogen , I. Nijs , and J. Lenoir . 2021. “Global Maps of Soil Temperature.” Global Change Biology 28: 3110–3144. 10.1111/gcb.16060 PMC9303923 34967074

[ece372515-bib-0081] Lomolino, M. V. 2001. “Elevation Gradients of Species‐Density: Historical and Prospective Views.” Global Ecology and Biogeography 10: 3–13.

[ece372515-bib-0082] López‐Angulo, J. , D. S. Pescador , A. M. Sánchez , M. A. K. Mihoč , L. A. Cavieres , and A. Escudero . 2018. “Determinants of High Mountain Plant Diversity in the Chilean Andes: From Regional to Local Spatial Scales.” PLoS One 13: 1–16. 10.1371/journal.pone.0200216 PMC6034847 29979767

[ece372515-bib-0083] Lüdecke, D. , D. Makowski , P. Waggoner , and I. Patil . 2020. “Performance: Assessment of Regression Models Performance.” R Package. CRAN.

[ece372515-bib-0084] Luebert, F. , and P. Pliscoff . 2006. Sinopsis bioclimática y vegetacional de Chile. Editorial Universitaria.

[ece372515-bib-0085] Magurran, A. E. 2004. Measuring Biological Diversity. Blackwell Publishing.

[ece372515-bib-0086] Mandakovic, D. , C. Aguado‐Norese , B. García‐Jiménez , et al. 2023. “Testing the Stress Gradient Hypothesis in Soil Bacterial Communities Associated With Vegetation Belts in the Andean Atacama Desert.” Environmental Microbiomes 18: 24. 10.1186/s40793-023-00486-w PMC10052861 36978149

[ece372515-bib-0087] Martinez Arbizu, P. 2020. “pairwiseAdonis: Pairwise Multilevel Comparison Using Adonis.”

[ece372515-bib-0088] Maurer, B. A. , and B. J. McGill . 2011. “Measurement of Species Diversity.” In Biological Diversity: Frontiers in Measurement and Assessment, edited by A. E. Magurran and B. J. McGill . Oxford Univ. Press; Oxford University Press.

[ece372515-bib-0089] MBN ‐ Ministero de Bienes Nacionales . 2019. “Información Territorial. DEM Alos Palsar Region Los Lagos.” https://www.ide.cl/index.php/imagenes‐y‐mapas‐base?start=20.

[ece372515-bib-0090] McCain, C. M. 2009. “Global Analysis of Bird Elevational Diversity.” Global Ecology and Biogeography 18: 346–360. John Wiley & Sons Ltd.

[ece372515-bib-0091] McCain, C. M. , and J. A. Grytnes . 2010. “Elevational Gradients in Species Richness.” In Encyclopedia of Life Sciences, 1–11. John Wiley & Sons Ltd.

[ece372515-bib-0092] McGlone, M. S. , C. H. Lusk , and J. J. Armesto . 2016. “Biogeography and Ecology of South‐Temperate Forests.” New Zealand Journal of Botany 54: 94–99.

[ece372515-bib-0093] Mestre, L. , M. Toro‐Manríquez , R. Soler , A. Huertas‐Herrera , G. Martínez‐Pastur , and M. V. Lencinas . 2017. “The Influence of Canopy‐Layer Composition on Understory Plant Diversity in Southern Temperate Forests.” Forest Ecosystems 4: 6.

[ece372515-bib-0094] Ministerio de Interior y Seguridad Pública . 2018. Amplíase el Parque Nacional Hornopirén, Ubicado en las Comunas de Cochamó y Hualaihué, Provincias de Llanquihue y Palena. Región de Los Lagos.

[ece372515-bib-0095] Miranda, A. , A. Altamirano , L. Cayuela , A. Lara , and M. González . 2017. “Native Forest Loss in the Chilean Biodiversity Hotspot: Revealing the Evidence.” Regional Environmental Change 17: 285–297.

[ece372515-bib-0096] MMA ‐ Ministero de Medio Ambiente . 2025. “Sistema de Información y Monitoreo de Biodiversidad. Parque Nacional “Hornopirén”. Límites y Cartografía.” https://simbio.mma.gob.cl/CbaAP/Details/956#limites.

[ece372515-bib-0097] Monge‐González, M. L. , D. Craven , T. Krömer , et al. 2020. “Response of Tree Diversity and Community Composition to Forest Use Intensity Along a Tropical Elevational Gradient.” Applied Vegetation Science 23: 69–79.

[ece372515-bib-0098] Morris, E. K. , T. Caruso , F. Buscot , et al. 2014. “Choosing and Using Diversity Indices: Insights for Ecological Applications From the German Biodiversity Exploratories.” Ecology and Evolution 4: 3514–3524. 25478144 10.1002/ece3.1155 PMC4224527

[ece372515-bib-0099] Mosseler, A. , J. A. Lynds , and J. E. Major . 2003. “Old‐Growth Forests of the Acadian Forest Region.” Environmental Reviews 11: 47–77.

[ece372515-bib-0100] Muller‐Landau, H. C. 2010. “The Tolerance–Fecundity Trade‐Off and the Maintenance of Diversity in Seed Size.” Proceedings of the National Academy of Sciences of the United States of America 107, no. 9: 4242–4247. 20160078 10.1073/pnas.0911637107 PMC2840174

[ece372515-bib-0101] Murphy, G. E. P. , and T. N. Romanuk . 2014. “A Meta‐Analysis of Declines in Local Species Richness From Human Disturbances.” Ecology and Evolution 4: 91–103. 24455164 10.1002/ece3.909 PMC3894891

[ece372515-bib-0103] Navarro, C. , C. Donoso , and V. Sandoval . 1999. “Los renovales de canelo.” In Silvicultura de los Bosques Nativos de Chile, edited by C. Donoso and A. Lara , 341–377. Editorial Universitaria.

[ece372515-bib-0104] Newbold, T. , L. N. Hudson , A. P. Arnell , et al. 2016. “Has Land Use Pushed Terrestrial Biodiversity Beyond the Planetary Boundary? A Global Assessment.” Science 353: 288–291. 27418509 10.1126/science.aaf2201

[ece372515-bib-0105] Newbold, T. , L. N. Hudson , S. L. L. Hill , et al. 2015. “Global Effects of Land Use on Local Terrestrial Biodiversity.” Nature 520: 45–50. 25832402 10.1038/nature14324

[ece372515-bib-0106] Nogués‐Bravo, D. , M. B. Araújo , T. Romdal , and C. Rahbek . 2008. “Scale Effects and Human Impact on the Elevational Species Richness Gradients.” Nature 453: 216–219. 18464741 10.1038/nature06812

[ece372515-bib-0107] O'Brien, E. 1998. “Water‐Energy Dynamics, Climate, and Prediction of Woody Plant Species Richness: An Interim General Model.” Journal of Biogeography 25: 379–398.

[ece372515-bib-0108] O'Brien, E. M. 1993. “Climatic Gradients in Woody Plant Species Richness: Towards an Explanation Based on an Analysis of Southern Africa's Woody Flora.” Journal of Biogeography 20: 181–198.

[ece372515-bib-0109] Ogden, J. 1995. “The Long‐Term Conservation of Forest Diversity in New Zealand.” Pacific Conservation Biology 2: 77–90.

[ece372515-bib-0110] Ohlemüller, R. , and J. B. Wilson . 2000. “Vascular Plant Species Richness Along Latitudinal and Altitudinal Gradients: A Contribution From New Zealand Temperate Rainforests.” Ecology Letters 3: 262–266.

[ece372515-bib-0111] Oksanen, J. , G. Simpson , F. Blanchet , et al. 2024. “vegan: Community Ecology Package.”

[ece372515-bib-0113] Pausas, J. G. , and M. P. Austin . 2001. “Patterns of Plant Species Richness in Relation to Different Environments: An Appraisal.” Journal of Vegetation Science 12: 153–166.

[ece372515-bib-0115] Peters, M. K. , A. Hemp , T. Appelhans , et al. 2016. “Predictors of Elevational Biodiversity Gradients Change From Single Taxa to the Multi‐Taxa Community Level.” Nature Communications 7: 1–11. 10.1038/ncomms13736 PMC5192166 28004657

[ece372515-bib-0114] Peters, M. K. , A. Hemp , T. Appelhans , et al. 2019. “Climate‐Land‐Use Interactions Shape Tropical Mountain Biodiversity and Ecosystem Functions.” Nature 568: 88–92. 30918402 10.1038/s41586-019-1048-z

[ece372515-bib-0116] Piazza, M.‐V. , L. A. Garibaldi , T. Kitzberger , and E. J. Chaneton . 2016. “Impact of Introduced Herbivores on Understory Vegetation Along a Regional Moisture Gradient in Patagonian Beech Forests.” Forest Ecology and Management 366: 11–22.

[ece372515-bib-0117] Pielou, E. C. 1975. Ecological Diversity. Wiley.

[ece372515-bib-0118] Piper, F. I. , A. Fajardo , G. Baeza , and L. A. Cavieres . 2019. “The Association Between a Nurse Cushion Plant and a Cluster Root‐Bearing Tree Species Alters the Plant Community Structure.” Journal of Ecology 107: 2182–2196.

[ece372515-bib-0119] Pliscoff, P. , M. J. Martínez‐Harms , and T. Fuentes‐Castillo . 2023. “Representativeness Assessment and Identification of Priorities for the Protection of Terrestrial Ecosystems in Chilean Patagonia.” In Conservation in Chilean Patagonia—Assessing the State of Knowledge, Opportunities, and Challenges, edited by J. C. Castilla , J. J. Armesto , M. J. Martínez‐Harms , and D. Tecklin . Pontificia Universidad Católica de Chile.

[ece372515-bib-0120] Pollmann, W. , and T. Veblen . 2004. “Nothofagus Regeneration Dynamics in South‐Central Chile: A Test of a General Model.” Ecological Monographs 74: 615–634.

[ece372515-bib-0121] Ponce, D. , P. Donoso , and C. Salas‐Eljatib . 2017. “Differentiating Structural and Compositional Attributes Across Successional Stages in Chilean Temperate Rainforests.” Forests 8: 329.

[ece372515-bib-0122] Quigley, M. F. , and W. J. Platt . 2003. “Composition and Structure of Seasonally Deciduous Forests in the Americas.” Ecological Monographs 73: 87–106.

[ece372515-bib-0123] R Core Team . 2024. R: A Language and Environment for Statistical Computing. R Foundation for Statistical Computing.

[ece372515-bib-0124] Raffaele, E. , T. Kitzberger , and T. Veblen . 2007. “Interactive Effects of Introduced Herbivores and Post‐Flowering Die‐Off of Bamboos in Patagonian Nothofagus Forests.” Journal of Vegetation Science 18: 371–378.

[ece372515-bib-0126] Rahbek, C. 1995. “The Elevational Gradient of Species Richness: A Uniform Pattern?” Ecography 18: 200–205.

[ece372515-bib-0125] Rahbek, C. , M. K. Borregaard , R. K. Colwell , et al. 2019. “Humboldt's Enigma: What Causes Global Patterns of Mountain Biodiversity?” Science 365: 1108–1113. 31515383 10.1126/science.aax0149

[ece372515-bib-0127] Ramírez‐Marcial, N. 2003. “Survival and Growth of Tree Seedlings in Anthropogenically Disturbed Mexican Montane Rain Forests.” Journal of Vegetation Science 14: 881–890.

[ece372515-bib-0128] Rodriguez, R. , C. Marticorena , D. Alarcón , et al. 2018. “Catálogo de las plantas vasculares de Chile.” Gayana. Botánica 75: 1–430.

[ece372515-bib-0129] Rodríguez‐Echeverry, J. , C. Echeverría , C. Oyarzún , and L. Morales . 2018. “Impact of Land‐Use Change on Biodiversity and Ecosystem Services in the Chilean Temperate Forests.” Landscape Ecology 33: 439–453.

[ece372515-bib-0130] Romdal, T. S. , and J.‐A. Grytnes . 2007. “An Indirect Area Effect on Elevational Species Richness Patterns.” Ecography 30: 440–448.

[ece372515-bib-0131] Rosenzweig, M. L. 1995. Species Diversity in Space and Time. Cambridge University Press.

[ece372515-bib-0132] Roswell, M. , J. Dushoff , and R. Winfree . 2021. “A Conceptual Guide to Measuring Species Diversity.” Oikos 130: 321–338.

[ece372515-bib-0133] Rüger, N. , Á. G. Gutiérrez , W. D. Kissling , J. J. Armesto , and A. Huth . 2007. “Ecological Impacts of Different Harvesting Scenarios for Temperate Evergreen Rain Forest in Southern Chile—A Simulation Experiment.” Forest Ecology and Management 252: 52–66.

[ece372515-bib-0134] Segovia, R. A. , and J. J. Armesto . 2015. “The Gondwanan Legacy in South American Biogeography.” Journal of Biogeography 42: 209–217.

[ece372515-bib-0135] Sharma, N. , M. D. Behera , A. P. Das , and R. M. Panda . 2019. “Plant Richness Pattern in an Elevation Gradient in the Eastern Himalaya.” Biodiversity and Conservation 28: 2085–2104.

[ece372515-bib-0136] Smith‐Ramírez, C. 2004. “The Chilean Coastal Range: A Vanishing Center of Biodiversity and Endemism in South American Temperate Rainforests.” Biodiversity and Conservation 13: 373–393.

[ece372515-bib-0137] Sommer, J. H. , H. Kreft , G. Kier , W. Jetz , J. Mutke , and W. Barthlott . 2010. “Projected Impacts of Climate Change on Regional Capacities for Global Plant Species Richness.” Proceedings of the Biological Sciences 277: 2271–2280. 20335215 10.1098/rspb.2010.0120 PMC2894901

[ece372515-bib-0138] Soto, D. P. , K. J. Puettmann , C. Fuentes , and D. F. Jacobs . 2019. “Regeneration Niches in *Nothofagus*‐Dominated Old‐Growth Forests After Partial Disturbance: Insights to Overcome Arrested Succession.” Forest Ecology and Management 445: 26–36.

[ece372515-bib-0139] Terborgh, J. , and J. S. Weske . 1975. “The Role of Competition in the Distribution of Andean Birds.” Ecology 56: 562–576.

[ece372515-bib-0140] van der Heijden, M. G. A. , J. N. Klironomos , M. Ursic , et al. 1998. “Mycorrhizal Fungal Diversity Determines Plant Biodiversity, Ecosystem Variability and Productivity.” Nature 396: 69–72.

[ece372515-bib-0141] Veblen, T. T. , C. Donoso , T. Kitzberger , and A. Rebertus . 1996. “Ecology of Southern Chilean and Argentinean Nothofagus Forests.” In The Ecology and Biogeography of Nothofagus Forests, edited by T. T. Veblen . Yale Univ. Press.

[ece372515-bib-0142] Vetaas, O. R. 2021. “Mountain Biodiversity and Elevational Gradients.” Frontiers of Biogeography 13, no. 3: 1–4.

[ece372515-bib-0143] Vetaas, O. R. , M. J. Steinbauer , C. Beierkuhnlein , et al. 2025. “Testing of Drivers for Plant Species Diversity Along Elevational Gradients in Seven Mountainous Islands in the Subtropics.” Journal of Vegetation Science 36, no. 4: e70053.

[ece372515-bib-0144] Vila, A. R. , and L. Borrelli . 2011. “Cattle in the Patagonian Forests: Feeding Ecology in Los Alerces National Reserve.” Forest Ecology and Management 261: 1306–1314.

[ece372515-bib-0145] Wallace, A. R. 1878. Tropical Nature, and Other Essays. Macmillan and Co.

[ece372515-bib-0146] Wang, X. , J. Fang , N. J. Sanders , P. S. White , and Z. Tang . 2009. “Relative Importance of Climate vs. Local Factors in Shaping the Regional Patterns of Forest Plant Richness Across Northeast China.” Ecography 32: 133–142.

[ece372515-bib-0147] Wassie, A. , F. J. Sterck , D. Teketay , and F. Bongers . 2009. “Effects of Livestock Exclusion on Tree Regeneration in Church Forests of Ethiopia.” Forest Ecology and Management 257: 765–772.

[ece372515-bib-0148] Watt, S. F. , D. M. Pyle , J. A. Naranjo , et al. 2011. “Holocene Tephrochronology of the Hualaihue Region (Andean Southern Volcanic Zone, ∼42°S), Southern Chile.” Quaternary International 246: 324–343.

[ece372515-bib-0149] Werenkraut, V. , and A. Ruggiero . 2011. “Quality of Basic Data and Method to Identify Shape Affect Richness‐Altitude Relationships in Meta‐Analysis.” Ecology 92, no. 1: 253–260. 21560695 10.1890/09-2405.1

[ece372515-bib-0159] Whittaker, R. H. 1972. “Evolution and Measurement of Species Diversity.” Taxon 21: 213–251.

[ece372515-bib-0151] Wickham, H. 2016. ggplot2: Elegant Graphics for Data Analysis. Springer.

[ece372515-bib-0150] Wickham, H. , R. François , L. Henry , K. Müller , and D. Vaughan . 2023. “dplyr: A Grammar of Data Manipulation.” R Package Version 1.1.4.

[ece372515-bib-0152] Wiens, J. J. , and M. J. Donoghue . 2004. “Historical Biogeography, Ecology and Species Richness.” Trends in Ecology & Evolution 19: 639–644. 16701326 10.1016/j.tree.2004.09.011

[ece372515-bib-0153] Wing, S. L. , and L. D. Boucher . 1998. “Ecological Aspects of the Cretaceous Flowering Plant Radiation.” Annual Review of Earth and Planetary Sciences 26: 379–421.

[ece372515-bib-0154] Wirth, C. , C. Messier , Y. Bergeron , D. Frank , and A. Fankhänel . 2009. “Old‐Growth Forest Definitions: A Pragmatic View.” In Old‐Growth Forests, edited by C. Wirth , G. Gleixner , and M. Heimann , 11–33. Springer.

[ece372515-bib-0155] Zanne, A. E. , D. C. Tank , W. K. Cornwell , et al. 2014. “Three Keys to the Radiation of Angiosperms Into Freezing Environments.” Nature 506: 89–92. 24362564 10.1038/nature12872

[ece372515-bib-0156] Zellweger, F. , P. de Frenne , and D. Coomes . 2020. “Forest Microclimate Dynamics Drive Plant Responses to Warming.” Science 368: 772–775. 32409476 10.1126/science.aba6880

[ece372515-bib-0157] Zhang, Y. , H. Y. H. Chen , and A. Taylor . 2014. “Multiple Drivers of Plant Diversity in Forest Ecosystems.” Global Ecology and Biogeography 23: 885–893.

[ece372515-bib-0158] Zuur, A. F. , E. N. Ieno , N. Walker , A. A. Saveliev , and G. M. Smith . 2009. Mixed Effects Models and Extensions in Ecology With R. Springer.

