# Supplementary material for: Diversity–Elevation Relationships of Vascular Plants in Austral Temperate Ecosystems Are Strata Dependent
Source: Ecol Evol. 2025 Dec 19;15(12):e72515. doi: 10.1002/ece3.72515 (PMC12715418; doi:10.1002/ece3.72515)
Supplement: Supplementary file 1 — Appendix S1: ece372515‐sup‐0001‐AppendixS1.zip. [file ECE3-15-e72515-s001.zip › suppl_file.docx]

**Supplementary Information**

**Table S1**. List of observed species (names following Rodriguez et al., 2018).

| **Family** | **Genus** | **epithet** | **Life form** | **Biogeographical status** |
| --- | --- | --- | --- | --- |
| Apiaceae | *Azorella* | *ranunculus* | Herb | native |
| Araliaceae | *Raukaua* | *laetevirens* | Shrub or small tree | native |
| Aspleniaceae | *Asplenium* | *dareoides* | Herb | native |
| Asteraceae | *Senecio* | *acanthifolius* | Herb | native |
| Asteraceae | *Gamochaeta* | *andina* | Herb | endemic |
| Asteraceae | *Belloa* | *chilensis* | Herb | native |
| Asteraceae | *Nassauvia* | *dentata* | Herb | native |
| Asteraceae | *Perezia* | *pedicularidifolia* | Herb | native |
| Asteraceae | *Senecio* | *kingii* | Herb | native |
| Asteraceae | *Senecio* | *trifurcatus* | Herb | native |
| Asteraceae | *Lagenophora* | *hariotii* | Herb | native |
| Asteraceae | *Hypochaeris* | spp. |  |  |
| Asteraceae | *Senecio* | spp. |  |  |
| Asteraceae | *Baccharis* | *magellanica* | Shrub | native |
| Asteraceae | *Senecio* | *triodon* | Sub-shrub | native |
| Asteraceae | *Senecio* | *portalesianus* | Sub-shrub | native |
| Asteraceae | *Senecio* | *bipontinii* | Sub-shrub | endemic |
| Asteraceae | *Archdasyphyllum* | *diacanthoides* | Tree | native |
| Atherospermataceae | *Laureliopsis* | *philippiana* | Tree | native |
| Berberidaceae | *Berberis* | *montana* | Shrub | native |
| Berberidaceae | *Berberis* | *microphylla* | Shrub | native |
| Berberidaceae | *Berberis* | *ilicifolia* | Shrub or small tree | native |
| Blechnaceae | *Blechnum* | *microphyllum* | Herb | native |
| Blechnaceae | *Blechnum* | *penna-marina* | Herb | native |
| Blechnaceae | *Blechnum* | *magellanicum* | Shrub | native |
| Bromeliaceae | *Greigia* | *landbeckii* | Herb | endemic |
| Celastraceae | *Maytenus* | *disticha* | Shrub or sub-shrub | native |
| Celastraceae | *Maytenus* | *magellanica* | Tree | native |
| Columelliaceae | *Desfontainia* | *fulgens* | Shrub or small tree | native |
| Cunoniaceae | *Caldcluvia* | *paniculata* | Tree | native |
| Cunoniaceae | *Weinmannia* | *trichosperma* | Tree | native |
| Cupressaceae | *Fitzroya* | *cupressoides* | Tree | native |
| Cyperaceae | *Carex* | *caduca* | Herb | native |
| Dicksoniaceae | *Lophosria* | *quadripinnata* | Sub-shrub | native |
| Dryopteridaceae | *Megalastrum* | *spectabile* | Herb | native |
| Elaeocarpaceae | *Crinodendron* | *hookerianum* | Tree | endemic |
| Ericaceae | *Empetrum* | *rubrum* | Shrub | native |
| Ericaceae | *Gaultheria* | *mucronata* | Shrub | native |
| Ericaceae | *Gaultheria* | *phillyreifolia* | Shrub | native |
| Ericaceae | *Gaultheria* | *pumila* | Shrub | native |
| Escalloniaceae | *Escallonia* | *alpina* | Shrub | native |
| Escalloniaceae | *Escallonia* | *leucantha* | Shrub | native |
| Escalloniaceae | *Escallonia* | *rubra* | Shrub | native |
| Escalloniaceae | *Escallonia* | *rosea* | Shrub | native |
| Escalloniaceae | *Tribeles* | *australis* | Sub-shrub | native |
| Eucryphiaceae | *Eucryphia* | *cordifolia* | Tree | native |
| Euphorbiaceae | *Dysopsis* | *glechomoides* | Herb | native |
| Gesneriaceae | *Asteranthera* | *ovata* | Herb | native |
| Gesneriaceae | *Mitraria* | *coccinea* | Shrub | native |
| Gleicheniaceae | *Sticherus* | *quadripartitus* | Herb | native |
| Griseliniaceae | *Griselinia* | *ruscifolia* | Shrub | native |
| Grossulariaceae | *Ribes* | *magellanicum* | Shrub | native |
| Gunneraceae | *Gunnera* | *magellanica* | Herb | native |
| Hymenophyllaceae | *Hymenophyllum* | *dentatum* | Herb | native |
| Hymenophyllaceae | *Hymenophyllum* | *umbratile* | Herb | native |
| Hymenophyllaceae | *Hymenophyllum* | *peltatum* | Herb | native |
| Hymenophyllaceae | *Hymenophyllum* | *tortuosum* | Herb | native |
| Hymenophyllaceae | *Hymenophyllum* | *ferrugineum* | Herb | native |
| Hymenophyllaceae | *Hymenophyllum* | *pectinatum* | Herb | native |
| Hymenophyllaceae | *Hymenophyllum* | *seselifolium* | Herb | native |
| Hymenophyllaceae | *Hymenophyllum* | *plicatum* | Herb | native |
| Juncaceae | *Oxychloe* | *andina* | Herb | native |
| Lentibulariaceae | *Pinguicula* | *chilensis* | Herb | native |
| Luzuriagaceae | *Luzuriaga* | *radicans* | Sub-shrub | native |
| Luzuriagaceae | *Luzuriaga* | *marginata* | Sub-shrub | native |
| Lycopodiaceae | *Austrolycopodium* | *confertum* | Herb | native |
| Myrtaceae | *Luma* | *chequen* | Shrub or small tree | endemic |
| Myrtaceae | *Myrceugenia* | *chrysocarpa* | Shrub or small tree | native |
| Myrtaceae | *Amomyrtus* | *luma* | Tree | native |
| Myrtaceae | *Luma* | *apiculata* | Tree | native |
| Myrtaceae | *Myrceugenia* | *planipes* | Tree | native |
| Nothofagaceae | *Nothofagus* | *antarctica* | Tree | native |
| Nothofagaceae | *Nothofagus* | *betuloides* | Tree | native |
| Nothofagaceae | *Nothofagus* | *dombeyi* | Tree | native |
| Nothofagaceae | *Nothofagus* | *nitida* | Tree | native |
| Nothofagaceae | *Nothofagus* | *pumilio* | Tree | native |
| Onagraceae | *Fuchsia* | *magellanica* | Shrub | native |
| Orchidaceae | *Gavilea* | *lutea* | Herb | native |
| Orchidaceae | *Codonorchis* | *lessonii* | Herb | native |
| Orobanchaceae | *Euphrasia* | *flavicans* | Herb | endemic |
| Oxalidaceae | *Oxalis* | *valdiviensis* | Herb | native |
| Philesiaceae | *Philesia* | *magellanica* | Shrub | native |
| Plantaginaceae | *Ourisia* | *breviflora subsp. uniflora* | Herb | native |
| Poaceae | *Chusquea* | *culeou* | Herb | native |
| Poaceae | *Chusquea* | *montana* | Herb | native |
| Poaceae | *Poa* | *alopecurus subsp. fuegiana* | Herb | native |
| Podocarpaceae | *Podocarpus* | *nubigena* | Tree | native |
| Proteaceae | *Embothrium* | *coccineum* | Tree | native |
| Proteaceae | *Lomatia* | *ferruginea* | Tree | native |
| Rosaceae | *Rubus* | *geoides* | Herb | native |
| Rosaceae | *Rubus* | *radicans* | Herb | native |
| Rosaceae | *Acaena* | *antarctica* | Herb | native |
| Rosaceae | *Acaena* | *ovalifolia* | Herb | native |
| Rubiaceae | *Nertera* | *granadensis* | Herb | native |
| Salicaceae | *Azara* | *lanceolata* | Shrub or small tree | native |
| Valerianaceae | *Valeriana* | *lapathifolia* | Herb | native |
| Valerianaceae | *Valeriana* | *fonckii* | Herb | native |
| Verbenaceae | *Rhaphithamnus* | *spinosus* | Shrub | native |
| Violaceae | *Viola* | *reichei* | Herb | native |
| Winteraceae | *Drimys* | *andina* | Shrub | endemic |
| Winteraceae | *Drimys* | *winteri* | Tree | native |


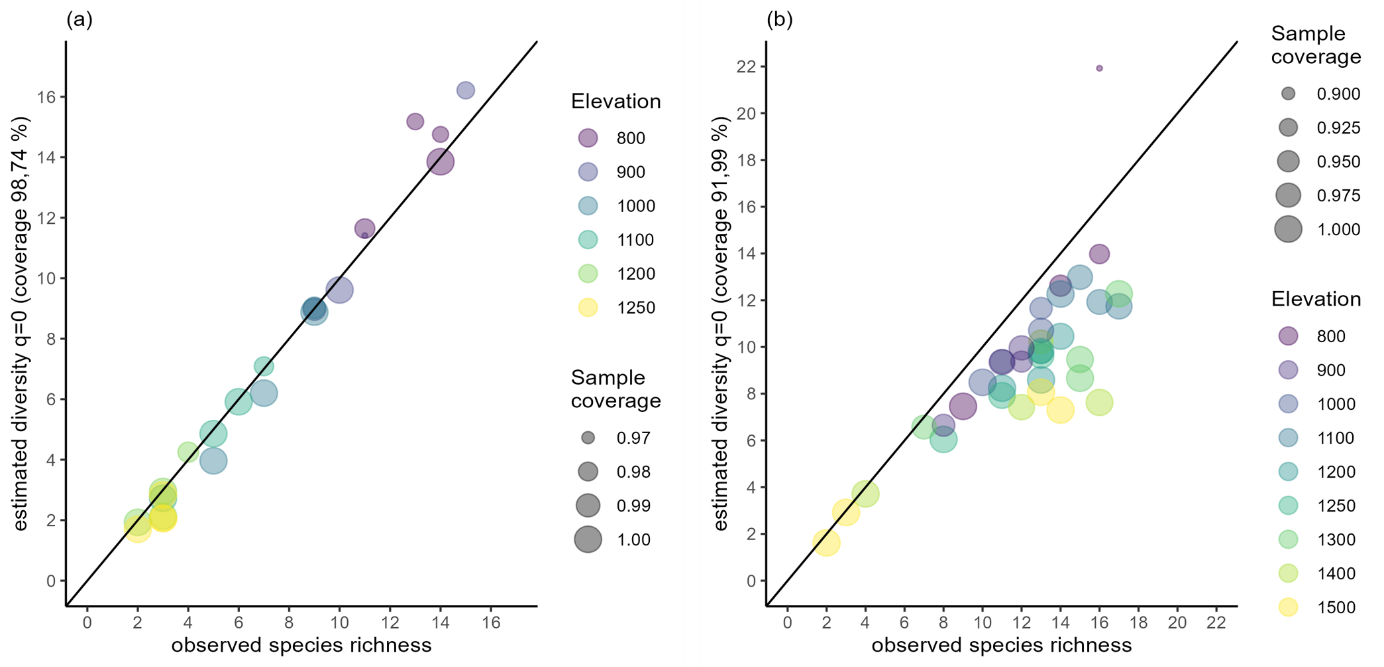


**Figure S2**: Observed species richness versus estimated diversity (*q=0*) of overstorey vegetation (**a**) and understorey vegetation (**b**) for the undisturbed part of the gradient (800 m - 1,250/1,500 m) in temperate rainforest and alpine scrub ecosystems at Hornopirén volcano, Chile. Point size is scaled by sample coverage.


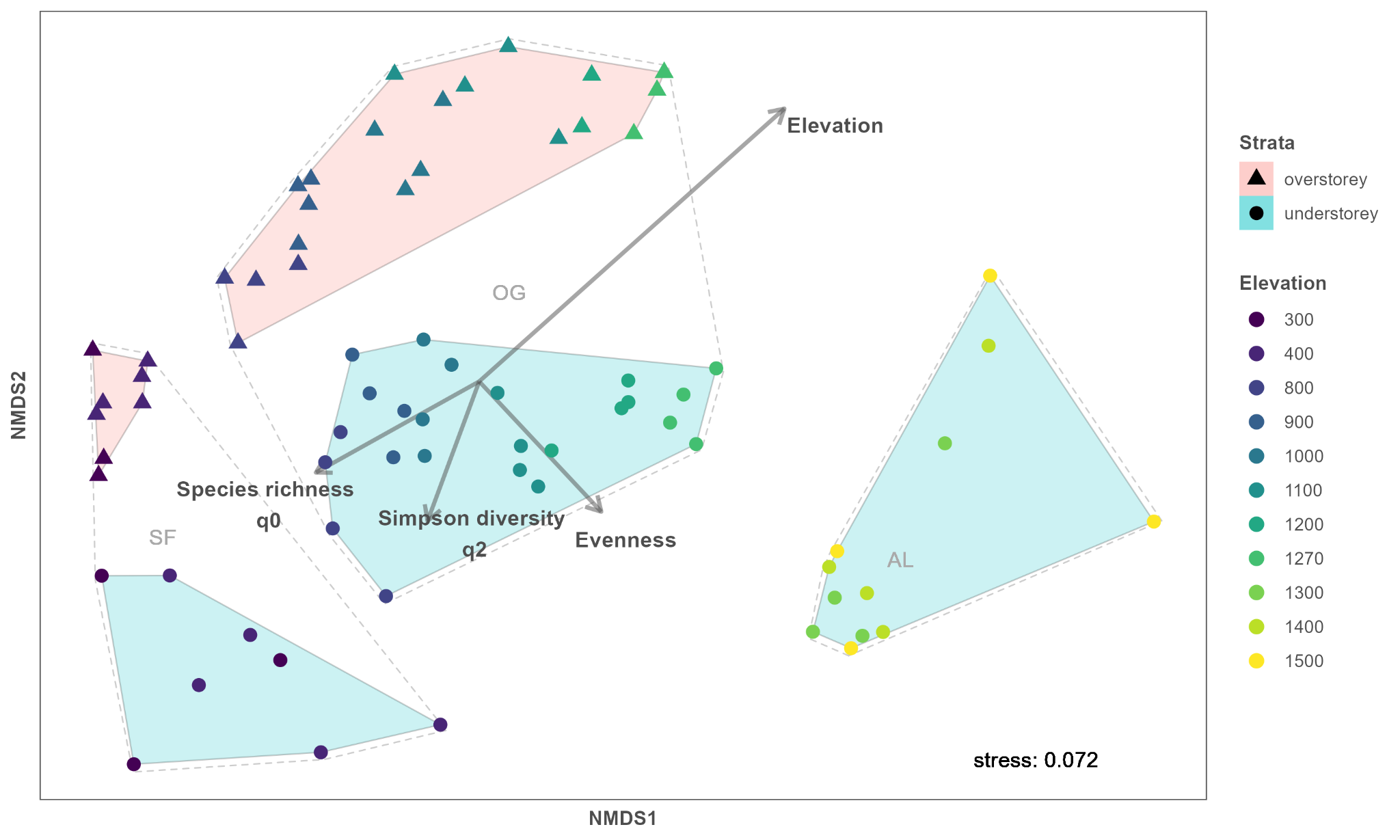


**Figure S3**.: Non-metric multidimensional scaling of species composition for overstorey strata (triangle symbols and pink convex hulls) and understorey strata (points and turqoise convex hulls) using Jaccard dissimilarity across the elevational gradient in temperate rainforest and alpine scrub ecosystems at Hornopirén volcano, Chile. Transparent hulls with dashed lines refer to the three segments of the elevational gradient: “SF” = Secondary Forest (300m & 400m), “OG” = Old Growth Forest (800m - 1270m), “AL” = Alpine Vegetation (1300m-1500m).

**Table S4** Results of the Permutational Multivariate Analysis of Variance (PERMANOVA) evaluating the effects of elevation (line 1-3), forest strata (line 4) and forest type (line 5-7) on community composition of the overstorey and understorey strata, respectively both strata together. Dissimilarity was estimated using incidence-based Jaccard dissimilarity and 999 permutations.

|  | **R²** | **F-value** | ***p*-value** |
| --- | --- | --- | --- |
| Overstorey vs. elevation | 0.32 | 13.85 | **0.001** |
| Understorey vs. elevation | 0.14 | 7.03 | **0.001** |
| Both strata vs. elevation | 0.12 | 10.34 | **0.001** |
| Overstorey vs. understorey | 0.11 | 7.76 | **0.001** |
| [Overstorey] Old-growth forest vs. secondary forest | 0.25 | 9.90 | **0.001** |
| [Understorey] Old-growth forest vs. secondary forest | 0.16 | 5.92 | **0.001** |
| [both forest strata] Old-growth forest vs. secondary forest | 0.14 | 9.73 | **0.001** |


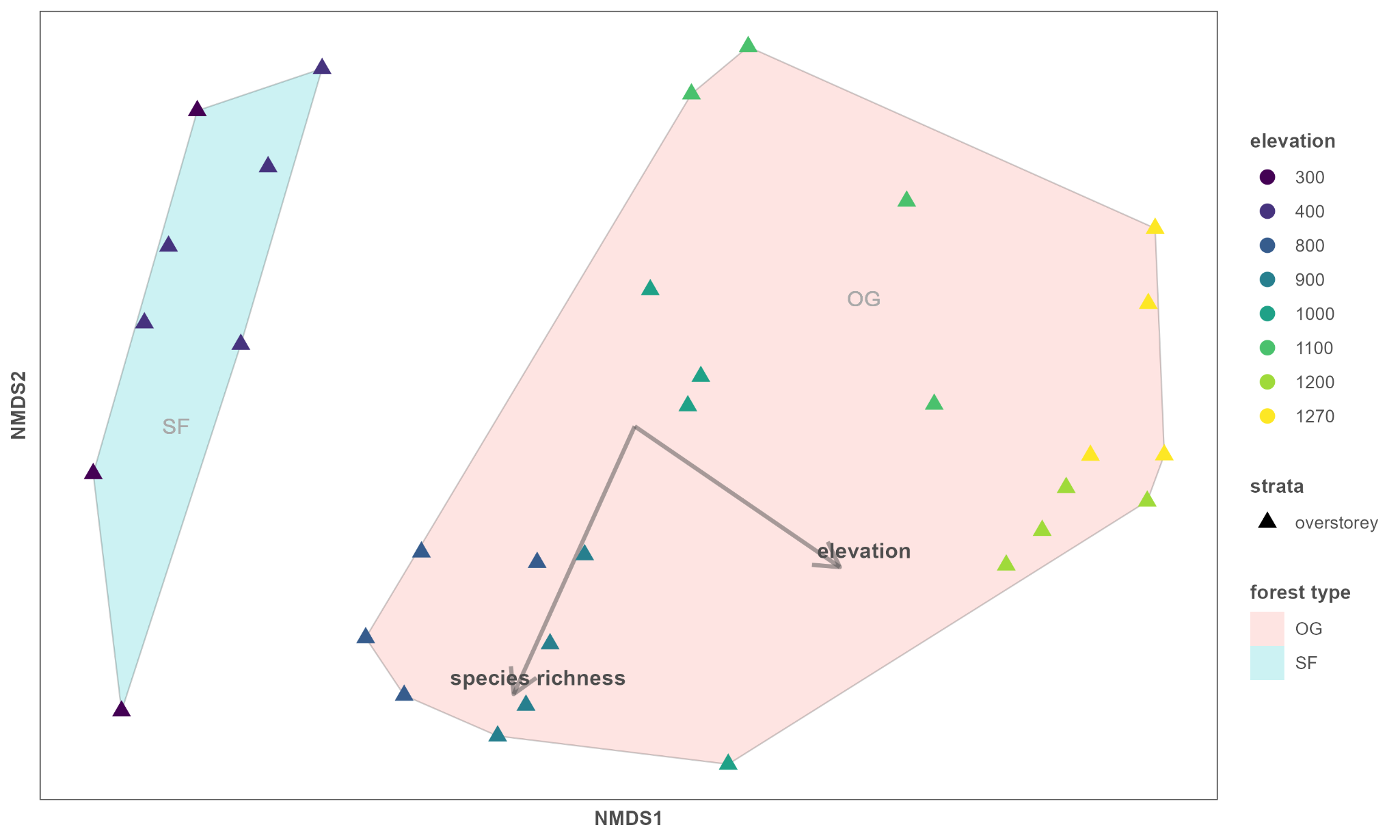


**Figure S5**.: Non-metric multidimensional scaling of species composition for the overstorey strata using Bray-Curtis dissimilarity across the elevational gradient in temperate rainforest at Hornopirén volcano, Chile. Convex hulls refer to the segments of the elevational gradient: “SF” = Secondary Forest (300m & 400m) and “OG” = Old Growth Forest (800m - 1270m).


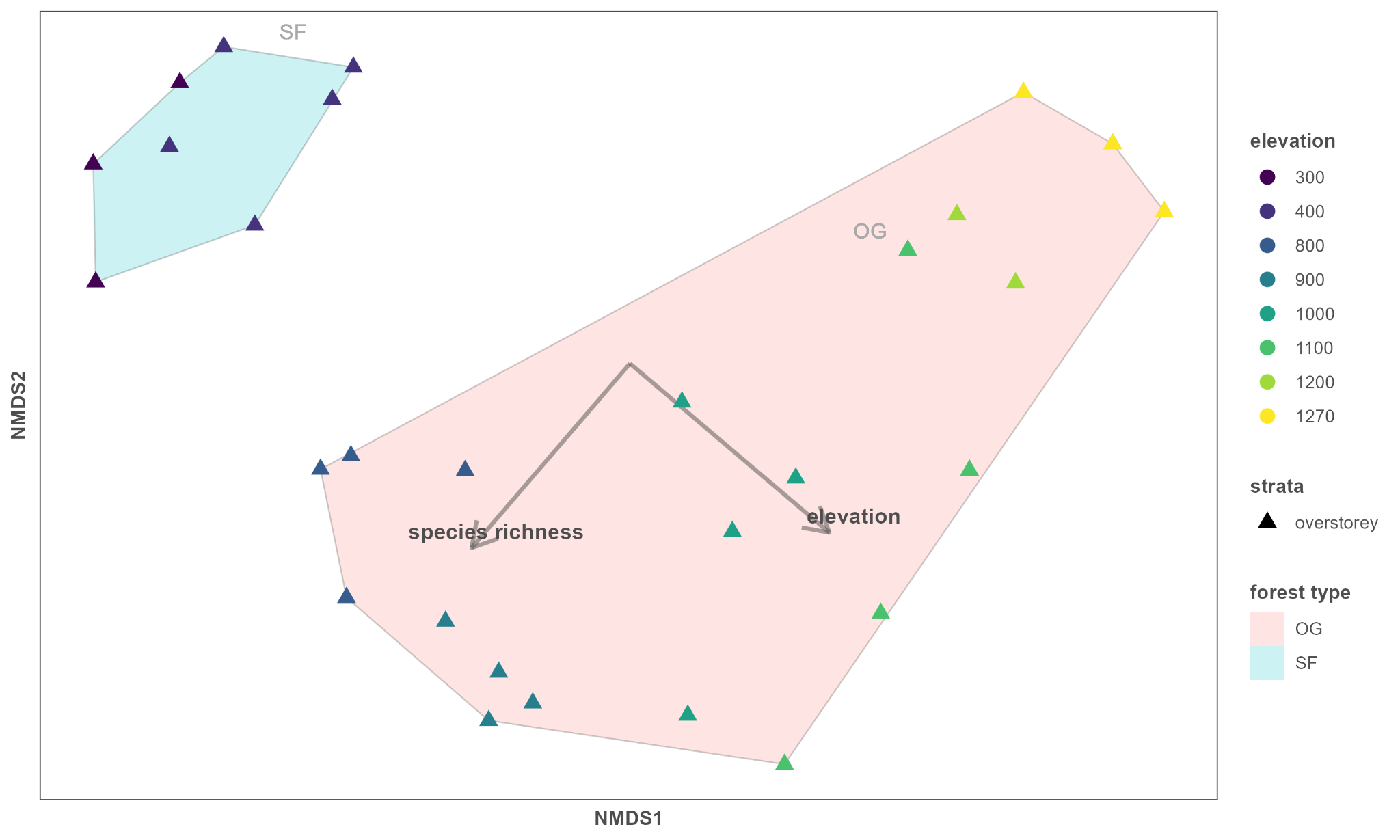


**Figure S6**.: Non-metric multidimensional scaling of species composition for the overstorey strata using Jaccard dissimilarity across the elevational gradient in temperate rainforest at Hornopirén volcano, Chile. Convex hulls refer to the segments of the elevational gradient: “SF” = Secondary Forest (300m & 400m), “OG” = Old Growth Forest (800m - 1270m).


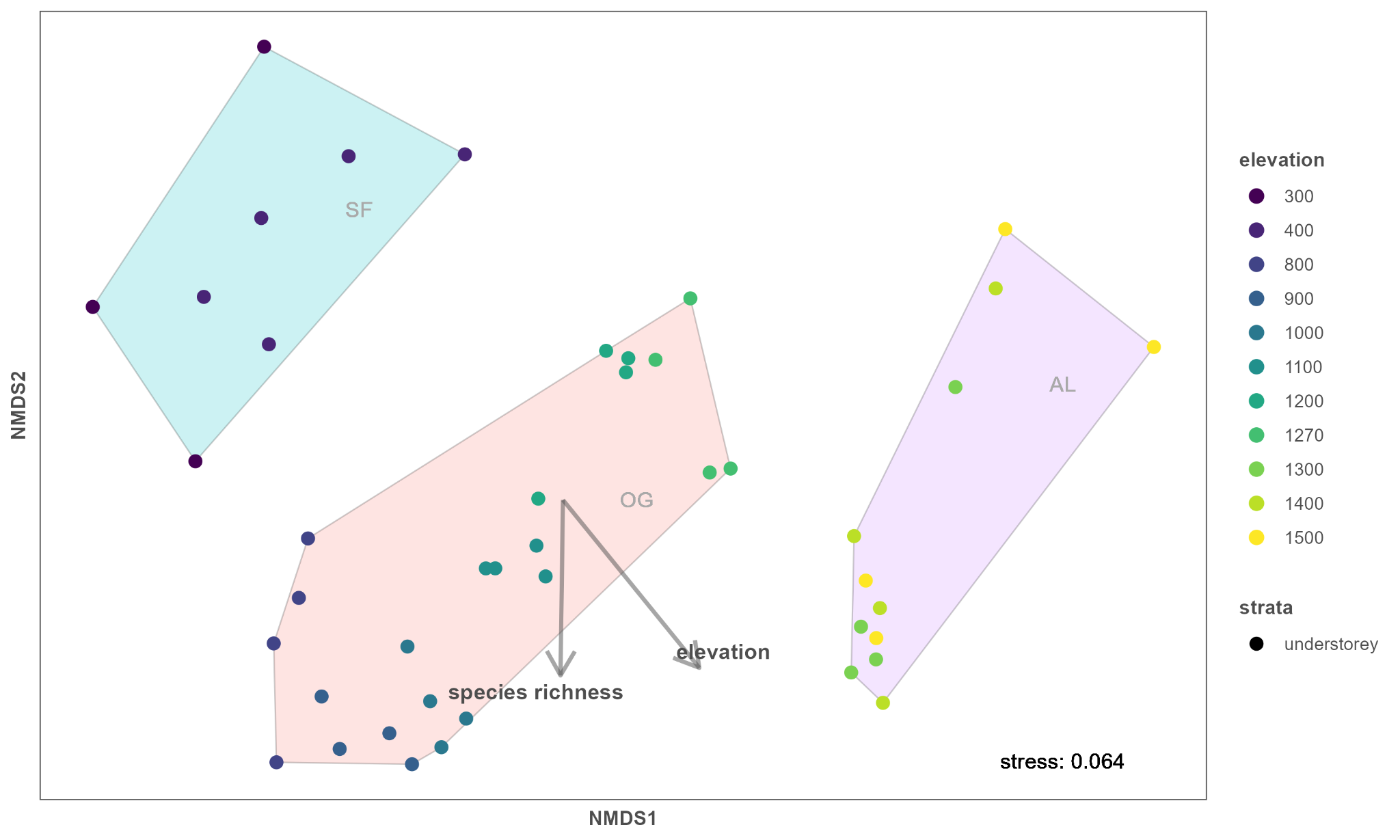


**Figure S7**.: Non-metric multidimensional scaling of species composition for the understorey strata using Bray-Curtis dissimilarity across the elevational gradient in temperate rainforest at Hornopirén volcano, Chile. Convex hulls refer to the segments of the elevational gradient: “SF” = Secondary Forest (300m & 400m), “OG” = Old Growth Forest (800m - 1270m), “AL” = Alpine vegetation (1,300m - 1,500m).


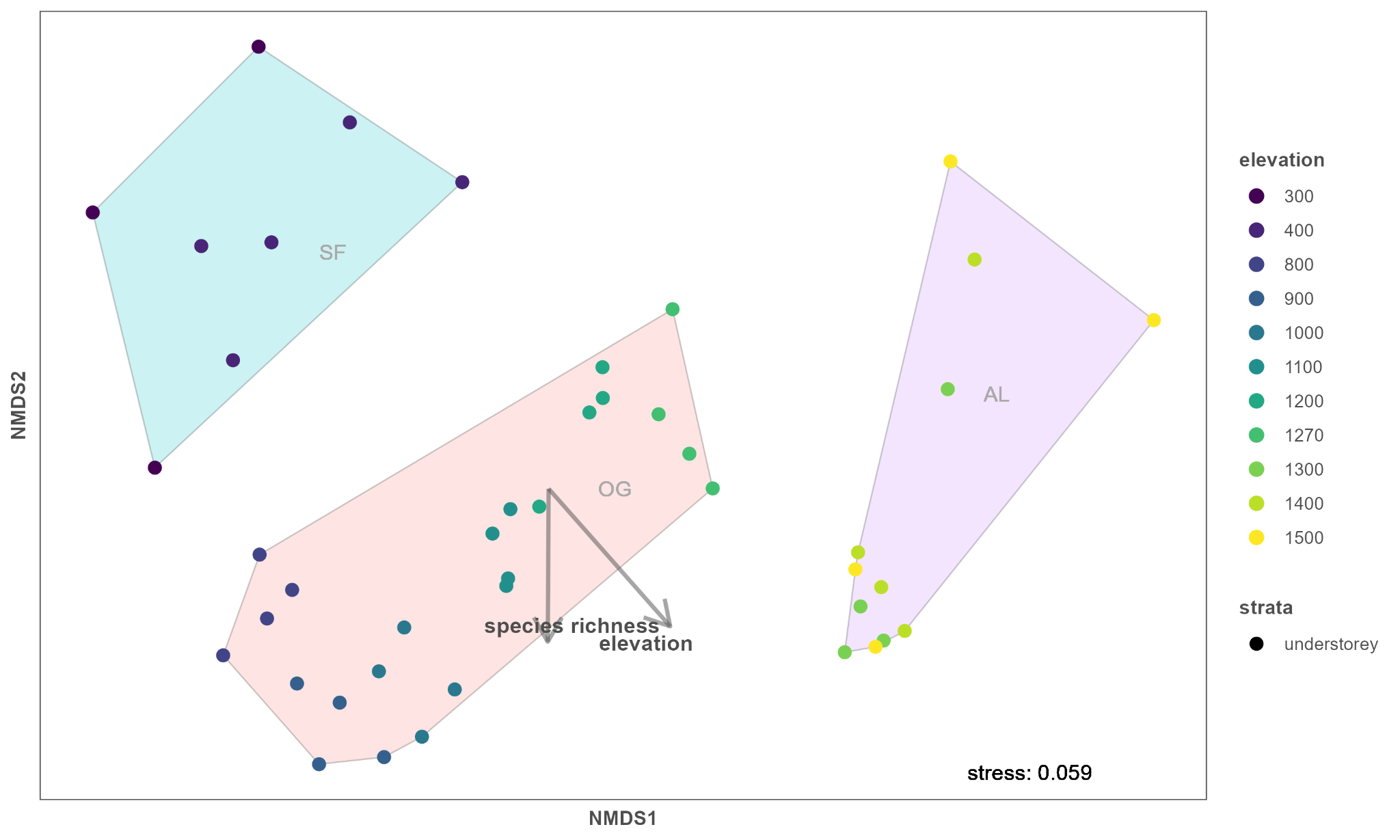


**Figure S8**.: Non-metric multidimensional scaling of species composition for the understorey strata using Jaccard dissimilarity across the elevational gradient in temperate rainforest at Hornopirén volcano, Chile. Convex hulls refer to the segments of the elevational gradient: “SF” = Secondary Forest (300m & 400m), “OG” = Old Growth Forest (800m - 1270m), “AL” = Alpine vegetation (1,300m - 1,500m).


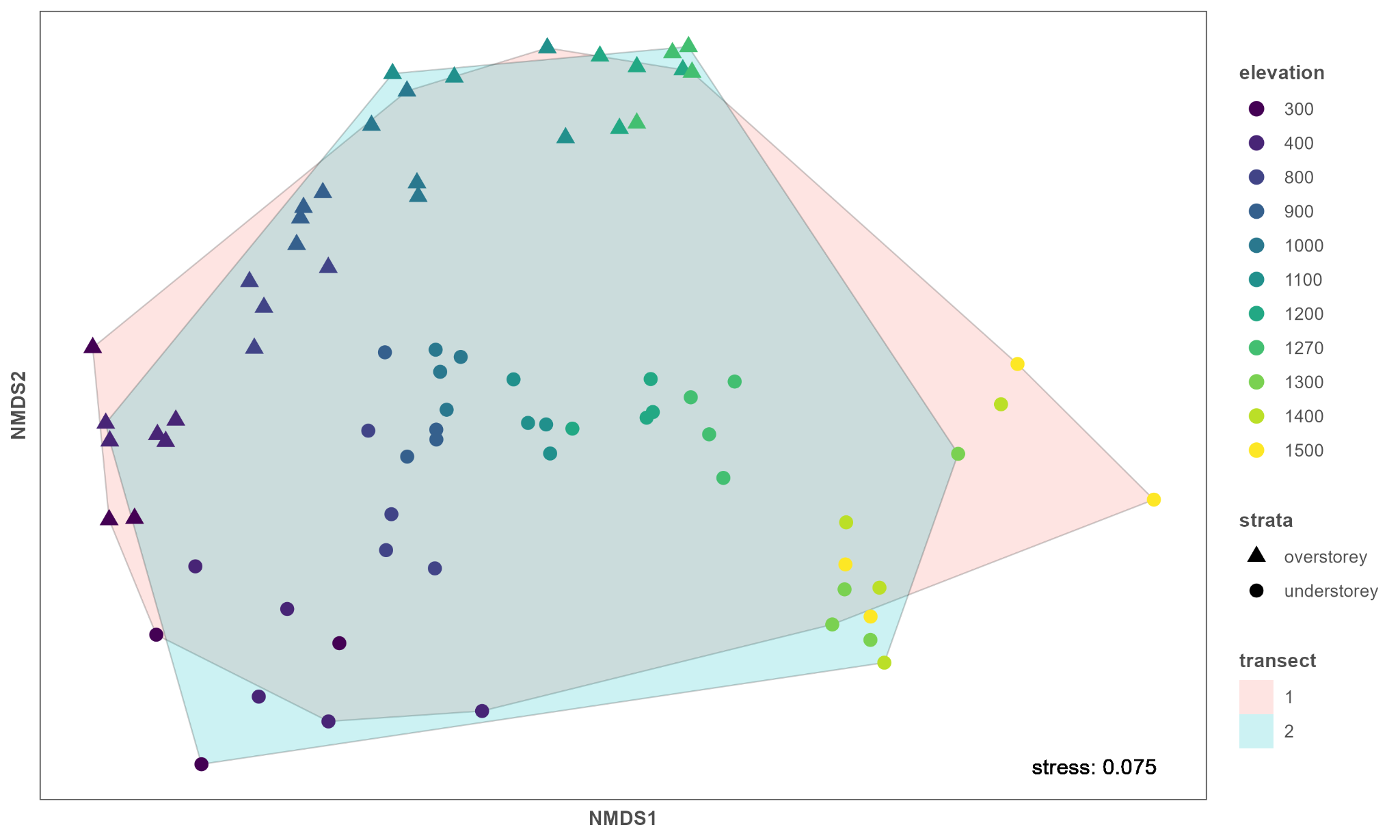
**Figure S9**.: Non-metric multidimensional scaling of species composition using Bray-Curtis dissimilarity between slope aspects in temperate rainforest and alpine scrub ecosystems at Hornopirén volcano, Chile. Convex hulls refer to Transect 1, respectively Transect 2. Species composition did not vary significantly between Transect 1 = North-east slope aspect and Transect 2 = South-west slope aspect. (PERMANOVA, p > 0.05).


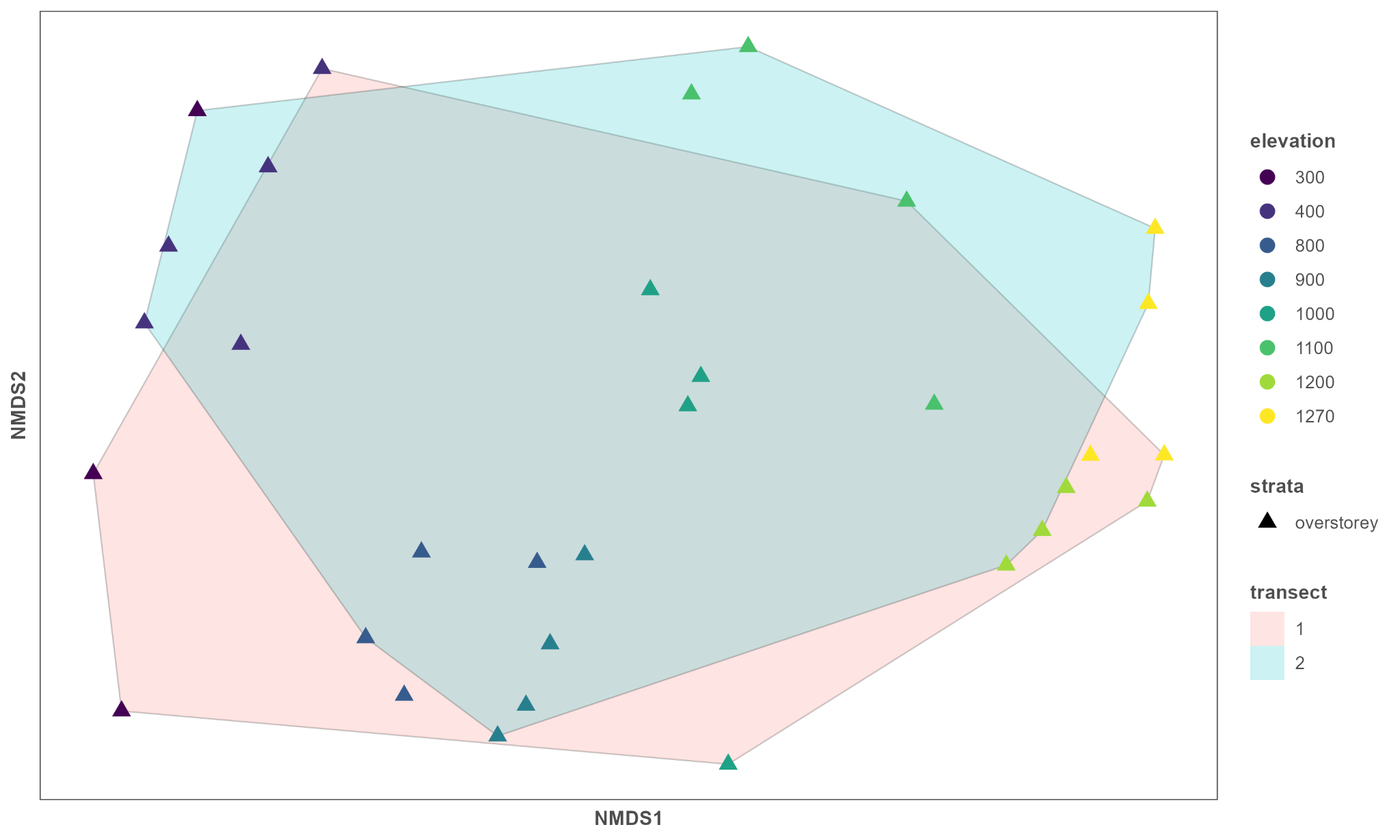
**Figure S10**.: Non-metric multidimensional scaling of species composition of overstorey vegetation using Bray-Curtis dissimilarity between slope aspects in temperate rainforest at Hornopirén volcano, Chile. Convex hulls refer to Transect 1, respectively Transect 2. Species composition did not vary significantly between Transect 1 = North-east slope aspect and Transect 2 = South-west slope aspect. (PERMANOVA, p > 0.05).


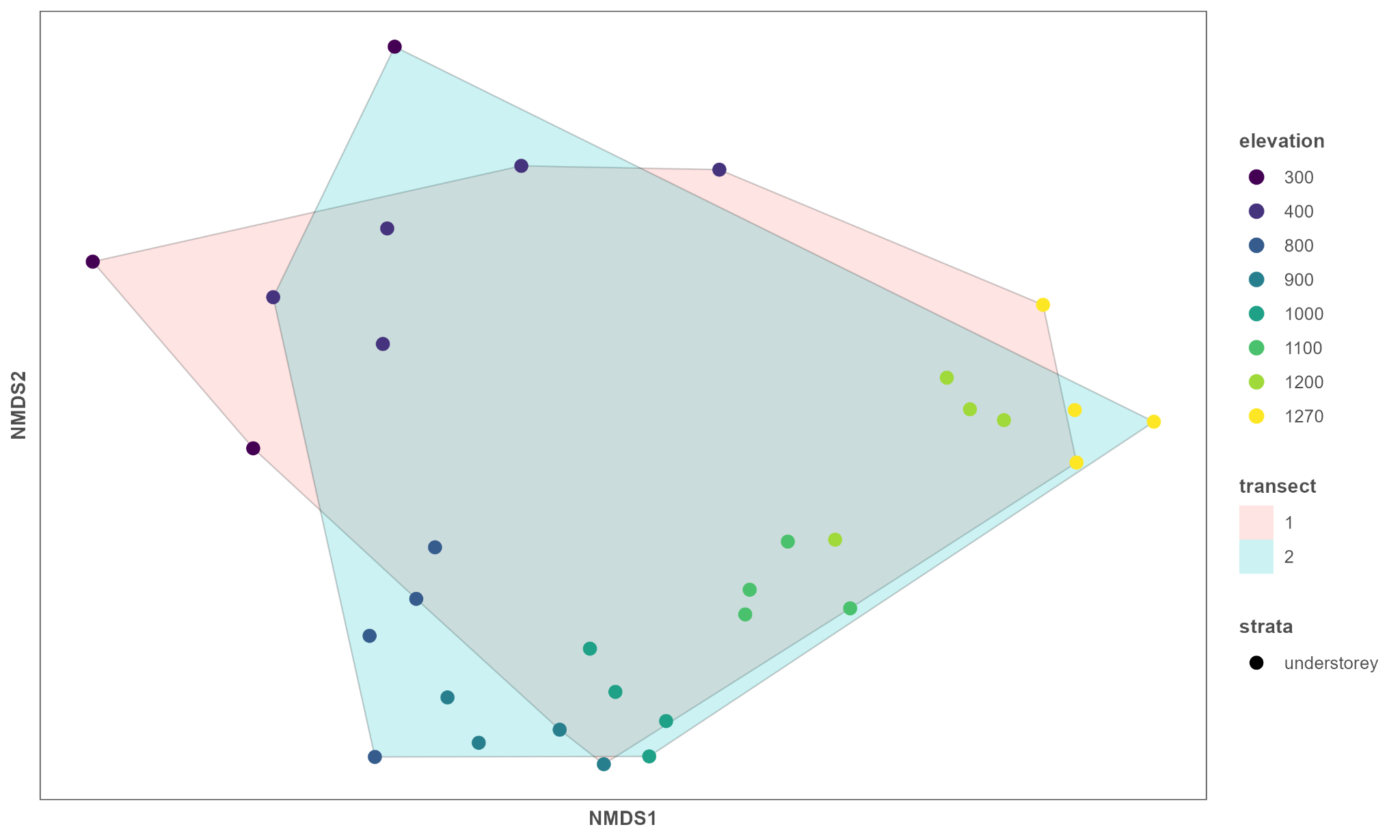
**Figure S11**.: Non-metric multidimensional scaling of species composition of understorey vegetation using Bray-Curtis dissimilarity between slope aspects in temperate rainforest and alpine scrub ecosystems at Hornopirén volcano, Chile. Convex hulls refer to Transect 1, respectively Transect 2. Species composition did not vary significantly between Transect 1 = North-east slope aspect and Transect 2 = South-west slope aspect. (PERMANOVA, p > 0.05).
